# Association between gestational environmental chemical mixtures and folate exposures with autistic behaviors in a Canadian birth cohort

**DOI:** 10.1097/EE9.0000000000000402

**Published:** 2025-06-11

**Authors:** Joshua D. Alampi, Bruce P. Lanphear, Amanda J. MacFarlane, Joseph M. Braun, Youssef Oulhote, Jillian Ashley-Martin, Tye E. Arbuckle, Aimin Chen, Gina Muckle, Lawrence C. McCandless

**Affiliations:** a Faculty of Health Sciences, Simon Fraser University, Burnaby, British Columbia, Canada; b Texas A&M Agriculture, Food, and Nutrition Evidence Center, Fort Worth, Texas; c Nutrition Research Division, Health Canada, Ottawa, Ontario, Canada; d Department of Epidemiology, Brown University, Providence, Rhode Island; e Department of Environmental Medicine and Public Health, Icahn school of medicine at Mount Sinai, New York City, New York; f Environmental Health Science and Research Bureau, Healthy Environments and Consumer Safety Branch, Health Canada, Ottawa, Ontario, Canada; g Department of Biostatistics, Epidemiology and Informatics, University of Pennsylvania Perelman School of Medicine, Philadelphia, Pennsylvania; h Centre Hospitalier Universitaire de Québec Research Centre and School of Psychology, Laval University, Québec City, Québec, Canada

**Keywords:** Environmental chemicals, Mixtures, Autism, Folate, Folic acid

## Abstract

**Background:**

Prenatal exposure to environmental chemicals may be associated with autism or autistic-like behaviors. Previous studies suggest that these associations are stronger when folic acid (FA) supplementation is lower.

**Methods:**

We used data from the Maternal-Infant Research on Environmental Chemicals study, a Canadian pregnancy and birth cohort (2008–2011). We considered five separate chemical mixtures (measured during the first trimester of pregnancy): metals, organochlorine pesticides, per- and polyfluoroalkyl substances (PFAS), polychlorinated biphenyls (PCBs), and persistent organic pollutants (POPs; including organochlorine pesticides, PFAS, PCBs, and one polybrominated diphenyl ether congener). Autistic-like behaviors were documented in 601 3–4-year-old children with the social responsiveness scale-2 (SRS-2), where higher T-scores denote more behaviors. We used quantile g-computation to estimate the mixture-SRS-2 associations and assessed whether gestational FA supplementation and plasma total folate concentrations modified these associations.

**Results:**

The PFAS mixture was associated with decreased SRS-2 T-scores (Ψ = −0.5; 95% confidence interval [CI] = −1.1, 0.1). The metal-SRS-2 associations were stronger in the positive direction among participants with high (>1,000 μg/d) FA supplementation (Ψ = 2.4; 95% CI = 0.8, 3.9) versus those with adequate (400–1,000 μg/d) supplementation (Ψ = −0.2; 95% CI = −1.1, 0.7) (p-interaction = 0.003). Plasma total folate concentrations similarly modified these associations (p-interaction = 0.01). The associations between the PFAS, PCB, and POP mixtures and SRS-2 T-scores were stronger in the positive direction among participants with low (<400 μg/d) versus adequate FA supplementation. This was not observed when assessing modification by plasma total folate concentrations.

**Conclusion:**

Our results suggest that the metal mixture is more strongly associated with autistic-like behaviors among participants with higher folate exposure, and the PFAS, PCB, and POP mixtures are more strongly associated with autistic-like behaviors among participants with low FA supplementation.

**What this study adds**

Understanding the impact of environmental chemicals on brain development, including their role in the development of autistic-like behaviors, is essential. Previous research suggests the relationship between chemicals and autism is stronger when folate consumption during pregnancy is lower. We examined the joint relationship between mixtures of chemical classes and autistic-like behaviors, and whether this relationship was modified by folate exposure during pregnancy. Our results suggest that metal concentrations are more strongly associated with increased autistic-like behaviors when gestational folate exposure is high. PFAS, polychlorinated biphenyls, and persistent organic pollutants may be more strongly associated with autistic-like behaviors when folic acid supplementation is low.

## Introduction

Environmental chemicals, including metals and persistent organic pollutants (POPs), have widespread health impacts, including on child neurodevelopment.^1,2^ POPs are chemicals that have long half-lives and can bioaccumulate.^3^ Examples include organochlorine pesticides, per- and polyfluoroalkyl substances (PFAS), polychlorinated biphenyls (PCBs), and polybrominated diphenyl ethers (PBDEs).^4^ Various studies suggest that metals,^5–9^ organochlorine pesticides,^9–14^ PFAS,^15–21^ PCBs,^9–14,22^ or PBDEs^11,13,23,24^ may be associated with autism or autistic-like behaviors, but findings are often mixed for individual chemicals. Relatively few studies have examined the effect of chemical mixtures, which may be used to examine the toxicity of entire classes.

The associations between metals and POPs and neurodevelopmental outcomes may be stronger when gestational folate exposure is lower. Research in humans, mice, and in vitro suggests that metals and POPs can induce oxidative stress, including oxidative damage to DNA,^25–27^ and impact in DNA methylation.^27–33^ In contrast, folate is associated with reduced oxidative stress,^34,35^ and contributes to DNA repair^35–37^ and cellular methylation potential.^36–38^ The neurotoxic effects of these chemicals may thus be diminished by gestational folate exposure. This is further supported by previous research indicating that gestational folate exposure may attenuate the associations of phthalate metabolites,^39,40^ air pollutants,^41^ lead,^42^ and self-reported household, commercial, or occupational pesticide exposure,^43^ with autism outcomes. However, this trend was not observed for PFAS^44^ or arsenic.^45^ To our knowledge, whether gestational folate exposure can modify the associations between PBDEs, PCBs, organochlorine pesticides, cadmium, or mercury and autism outcomes has not been assessed. Investigating ways to mitigate the neurotoxic impacts of environmental chemicals is of great public health importance, given the difficulty of entirely eliminating exposure.^46,47^

In this article, we assessed the associations between mixtures of various chemical classes and childhood autistic behaviors using data from the Maternal-Infant Research on Environmental Chemicals (MIREC) study, a pan-Canadian cohort. We also examined whether these associations were modified by folic acid (FA) supplementation, plasma total folate concentrations, and child sex.

## Methods

### Study participants

We used data from the MIREC study, a pregnancy and birth cohort that enrolled women in 10 Canadian cities from 2008 to 2011.^48^ Eligibility criteria were as follows: ≥18 years old, <14 weeks gestation, able to communicate in French or English, planning on delivering at a local hospital, consenting to cord blood collection, not having medical complications, and not carrying a fetus with known abnormalities. We approached 8,716 individuals; 1983 met the inclusion criteria and 1862 had a singleton live birth. Neurodevelopmental outcomes were assessed in a convenience subsample of 610 3- to 4-year-old children from six of the 10 cities.^49^ We restricted our analysis to the 601 mother-child pairs for which autistic-like behaviors were documented (Figure S1; https://links.lww.com/EE/A353).

Health Canada Research Ethics Board and the Institutional Review Boards of Simon Fraser University, all recruitment sites, and Centre Hospitalier Universitaire Sainte-Justine Research Centre approved the MIREC study. All participants provided written consent for their own and their children’s participation in this study.

### Autistic-like behaviors

The preschool-aged version of the social responsiveness scale-2 (SRS-2) 65-item questionnaire was completed by one parent during an in-home visit.^50^ A child’s total score (T-score) is calculated based on this questionnaire, where higher T-scores denote a higher degree of communication problems and repetitive/nonreciprocal behaviors. A T-score between 60 and 75 denotes mild to moderate autistic-like traits, and a T-score >75 denotes severe autistic-like traits.^50^ The SRS-2 is a sensitive, valid, and reliable tool for documenting autistic-like behaviors.^51,52^

### Biomarkers of chemical exposure

We restricted our analysis to chemicals that had a concentration greater than the limit of detection (LOD) in at least 60% of samples. Chemical concentrations were assessed during the first trimester of pregnancy (6–13 weeks of gestation). Blood-metal concentrations were assessed with inductively coupled plasma-mass spectrometry.^53^ Plasma-POP concentrations were assessed with mass spectrometry and gas chromatography.^54^ All samples were temporarily stored at −20 °C at local study sites before long-term storage at −80 °C. We divided the concentrations of organochlorine pesticides, PCBs, and PBDEs by maternal total lipid concentrations.^54,55^ Biomarker concentrations below the LOD were estimated using single imputation. The mean and standard deviation of a truncated lognormal distribution were calculated for these variables using existing data (with below-LOD concentrations being temporarily assigned LOD/$\sqrt{2}$). Concentrations below the LOD were randomly assigned from this distribution.^56^

### Covariates

We created a directed acyclic graph to identify variables that may confound the relationship between gestational exposure to environmental chemical mixtures and child SRS-2 T-scores (Figure S2; https://links.lww.com/EE/A353).^57^ We identified the following confounding variables: FA supplementation (described below), household income, maternal age at enrollment, maternal education, maternal race (do they self-identify as White or not), maternal relationship status (do they live with their partner), maternal smoking during pregnancy, parity, study site, and year of enrollment.^58–66^ To improve precision, we also controlled for variables that could be associated with SRS-2 T-scores,^67^ including: child sex, child age at SRS-2 assessment, and home observation for measurement of the environment (HOME) score.^68–70^ Covariate information was collected via a questionnaire administered during enrollment; child sex was extracted from medical charts. HOME scores were determined by in-home observations and interviews with the child’s caregiver(s) by trained interviewers at the same time as the SRS-2 assessment, and reflect the quality of the stimulation provided to the child.^70^

### Gestational folic acid supplementation

FA supplementation was primarily measured using a structured survey conducted at approximately 16 weeks gestation, where participants were asked to list all “vitamins, minerals, homeopathic medicines, and/or natural products” they had taken in the past 30 days (n = 492). When participants did not indicate any FA supplementation on the 30-day recall form, we cross-referenced this with the (otherwise identical) 24-hour recall form (n = 5). If a participant reported FA supplementation on the 24-hour but not the 30-day recall form, then we used the 24-hour recall form (which appears first) (n = 4). If a participant did not submit the 30-day recall form, we used the 24-hour recall form (n = 9). If neither of these forms was submitted, we used data from the baseline questionnaire (n = 74). We cross-referenced the 24-hour recall form with the baseline questionnaire to fill in missing details on the 24-hour recall form (n = 17). Unlike the 30-day or 24-hour recall forms, the baseline questionnaire was completed upon study enrollment (6–13 weeks gestation) and queried “vitamin/mineral supplementation” in the past 3 months. We assumed that FA supplementation was constant throughout pregnancy. See Alampi et al. ^42^ for further details.

We categorized participants’ FA intake from supplements as <400, 400–1,000, or ≥1,000 μg/d. This corresponds to the recommended supplementation for individuals who could become pregnant or are pregnant/lactating (400 μg/d),^71,72^ and the allowable maximum for over-the-counter prenatal supplements in Canada (1,000 μg/d).^72,73^

### Plasma total folate concentrations

Folate vitamers were measured using liquid chromatography-tandem mass spectrometry, as previously described.^74^ We calculated first-trimester (6–13 weeks gestation) plasma total folate concentrations by summing five folate vitamers: tetrahydrofolate (THF), 5-formyl-THF, 5,10-methenyl-THF, unmetabolized FA, and 5-methyl-THF. Most samples were not fasting samples, which may introduce measurement error.^53,74,75^

Because approximately 10% and 20% of our sample reported a daily FA supplementation of <400 or >1,000 μg, we selected the 10th and 80th percentiles of plasma total folate concentrations as cutoffs. We assumed that lower and higher FA supplementation would reflect lower and higher plasma total folate concentrations.

### Statistical analyses

First, we assessed the covariate-adjusted associations between each individual chemical (with concentrations log_2_-transformed) and SRS-2 T-scores using linear regression models. Next, we used quantile g-computation to assess the joint effects of five chemical mixtures on child SRS-2 T-scores. Quantile g-computation reports the estimated difference in SRS-2 T-score when simultaneously increasing the concentrations of all chemicals in a given mixture by one quartile.^76^ We also assessed the associations between these mixtures and SRS-2 T-scores among participants in the lowest, middle (reference), and highest categories of FA supplementation and plasma total folate concentration, and among male and female children. We selected the following chemical mixtures a priori: metals (four chemicals), organochlorine pesticides (four congeners), PCBs (four congeners), PFAS (three chemicals), and POPs (12 chemicals, including all organochlorine pesticides, PCBs, PFAS, and one PBDE congener). We included a “POP” mixture because a previous MIREC-based study identified “high POP” and “low POP” profiles using unsupervised machine learning techniques.^77^ This implies that participants who had high concentrations of one POP tended to have high concentrations of other POPs, and vice versa.

In all analyses, we addressed missing data (chemical concentrations, plasma total folate concentrations, education, income, and HOME scores) using multiple imputation by chained equations with 10 multiply imputed datasets.^78,79^ Note that we did not pool the quantile g-computation weights and instead reported weights from the first imputation, see Appendix 1; https://links.lww.com/EE/A353.^76^ We also implemented inverse probability weighting (IPW) in all analyses to address possible selection bias associated with the SRS-2 being conducted in a convenience subsample of the original MIREC cohort.^80,81^ Using data from all participants with a singleton live birth (n = 1,862), we estimated the probability of inclusion in the convenience subsample with a logistic regression model with the following covariates: household income, maternal age at enrollment, education, race, smoking during pregnancy, study site, year of enrollment, and whether the mother lives with their partner. Covariates were selected if (i) the covariate is a confounder or predictive of SRS-2 T-scores (Figure S2; https://links.lww.com/EE/A353) and (ii) we determined that the covariate was predictive of non-follow up (*P*-value for *χ*^2^ test <0.1 when comparing participants with [n = 601] and without [n = 1,261] SRS-2 T-scores, see Table S1; https://links.lww.com/EE/A353).^80^ We used the stabilized inverse of each participants’ estimated probability of inclusion in the convenience sample as the weights in all analyses.^80^ All analyses were carried out only for the n = 601 participants who had SRS-2 T-scores. We utilized the “qgcomp,”^76^ “qgcompint,”^76^ and “mice”^79^ packages and R version 4.4.3 for all analyses.

### Exploratory and sensitivity analyses

Quantile g-computation assumes directional homogeneity, that the associations between all chemicals in a mixture and the outcome have the same sign.^76^ However, if the individual chemicals in a mixture have contradictory associations with the outcome, the associations may cancel each other out. Thus, quantile g-computation may characterize a mixture as having no or small associations with the outcome even if the mixture includes chemical(s) that are strongly associated with the outcome.^82^ To address this limitation, we estimated the associations between the five chemical mixtures and SRS-2 T-scores with weighted quantile sum regression using the “gWQS” package.^83^ This mixture method allows one to separately estimate the positive and negative effects of a mixture on the outcome, and may be better at characterizing the effects of a mixture when the directional homogeneity assumption is not met.^82,84^ For this analysis, we averaged the weights generated across 10 multiply imputed datasets, see Appendix 1; https://links.lww.com/EE/A353.

To further investigate our findings, we assessed whether individual chemicals were modified by FA supplementation and plasma total folate concentrations. We also assessed whether having “high” (defined a priori as >80th percentile) unmetabolized FA concentrations also modified the mixture-SRS-2 associations. Next, we assessed the mixture-SRS-2 associations while additionally controlling for gestational fish consumption, which may influence metal and POP concentrations.^3,5,17^ Finally, we compared the mixture results with and without IPW to probe the direction of biases.

## Results

### Descriptive statistics

Most participants in our sample lived with their partner (97%), self-identified as being White (90%), were noncigarette smokers (92%), and had a university degree (67%) (Table 1). Eleven children (2%) had a SRS-2 T-score ≥60, indicating at least mild autistic-like behaviors.^50,85^ SRS-2 T-scores were higher in male children, children whose mothers smoked during pregnancy, children from lower-income households, and children with lower HOME scores (Table 1).

**Table 1.** Sociodemographic characteristics of participants, MIREC study, Canada, 2008–2011 (n = 601)

Variable	n (%)	Mean SRS-2 T-score (SD)
All	601 (100.0)	45.3 (6.1)
Child sex		
Male	290 (48.3)	46.6 (6.5)
Female	311 (51.7)	44.2 (5.3)
Maternal age at enrollment (years)		
18–29	132 (22.0)	46.7 (5.6)
30–35	292 (48.6)	45.3 (6.4)
≥36	177 (29.5)	44.4 (5.8)
Living with partner		
Yes	580 (96.5)	45.2 (6.0)
No	21 (3.5)	48.8 (7.3)
Maternal race		
White	539 (89.7)	45.1 (6.1)
Other	62 (10.3)	47.1 (5.7)
Education level		
High school or less	30 (5.0)	47.8 (7.1)
College or trade school	167 (27.8)	46.2 (5.8)
Undergraduate university degree	239 (39.8)	45.4 (6.6)
Graduate university degree	163 (27.1)	44.0 (5.1)
Missing	2 (0.3)	
Annual household income ($CAD)		
≤$40,000	61 (10.1)	47.9 (6.1)
$40,001–$80,000	173 (28.8)	46.3 (6.3)
$80,001–$100,000	116 (19.3)	45.1 (6.1)
>$100,000	231 (38.4)	44.1 (5.6)
Missing	20 (3.3)	
Parity		
Nulliparous	261 (43.4)	46.1 (6.1)
Uniparous	251 (41.8)	44.6 (5.7)
Multiparous	89 (14.8)	45.1 (6.9)
HOME score		
≥48 (median)	323 (53.7)	44.3 (5.3)
<48 (median)	260 (43.3)	46.7 (6.7)
Missing	18 (3.0)	
Smoked during pregnancy		
Yes^a^	47 (7.8)	48.2 (6.8)
No	554 (92.2)	45.1 (6.0)
Year of enrollment		
2008	10 (1.7)	48.0 (5.0)
2009	187 (31.1)	45.2 (6.1)
2010	381 (63.4)	45.3 (6.1)
2011	23 (3.8)	46.0 (6.2)
Child age at SRS assessment		
≥40 months (median)	322 (53.6)	45.0 (6.5)
<40 months (median)	279 (46.4)	45.8 (5.5)
First-trimester fish consumption		
0–2 times per month	220 (36.6)	45.8 (6.1)
3–7 times per month	209 (34.8)	45.3 (6.3)
≥8 times per month	169 (28.1)	44.9 (5.7)
Missing	3 (0.5)	
Folic acid supplementation^b^		
<400 µg per day	34 (5.7)	45.4 (5.5)
400–1,000 µg per day	423 (70.4)	45.3 (6.2)
>1,000 µg per day	144 (24.0)	45.4 (6.0)
Plasma total folate concentrations^c^		
<10th percentile (65.6 nmol/l)	48 (8.0)	45.5 (6.6)
10th–80th percentile	409 (68.1)	45.3 (6.2)
>80th percentile (125 nmol/l)	116 (19.3)	45.4 (5.6)
Missing	28 (4.7)	
Unmetabolized folic acid concentrations		
≤80th percentile	461 (76.7)	45.6 (6.4)
>80th percentile (13.6 nmol/l)	112 (18.6)	44.5 (4.9)
Missing	28 (4.7)	

a Includes current smokers and individuals who quit during pregnancy. “Nonsmoker” includes participants who did not smoke and former smokers.

b Folic acid supplementation was primarily measured via a survey conducted at 16 weeks gestation, which queried intake in the past 30 days. We also used data from the 24-hour recall version of this survey and a questionnaire completed at study enrollment (6–13 weeks gestation).

c Sum of 5-formyl-THF, 5–10-methylene-THF, THF, UMFA, 5-methyl-THF.

CAD indicates Canadian dollar; HOME, home observation for measurement of the environment; MIREC, Maternal-Infant Research on Environmental Chemicals study; SD, standard deviation; SRS, social responsiveness scale; THF, tetrahydrofolate; UMFA, unmetabolized folic acid.

**Figure 1.**
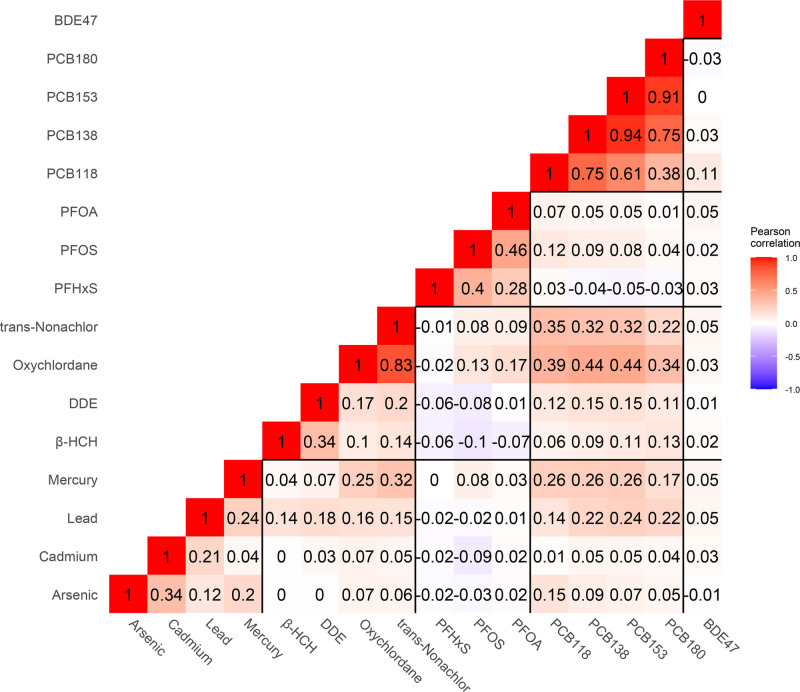
Pearson correlation matrix between individual chemicals, the MIREC study, Canada, 2008–2011 (n = 601). BDE indicates brominated diphenyl ether; DDE, dichlorodiphenyldichloroethylene; MIREC, Maternal-Infant Research on Environmental Chemicals study; PCB, polychlorinated biphenyl; PFHxS, Perfluorohexanesulfonic acid; PFOA, Perfluorooctanoic acid; PFOS, Perfluorooctanesulfonic acid; β-HCH, β-hexachlorocyclohexane.

All metals, all PFAS chemicals, two out of four organochlorine pesticide congeners, and three out of four PCB congeners were detectable in more than 90% of our samples (Table 2). Most PCB congeners were strongly correlated with each other (ρ = 0.38–0.94). PFAS chemicals were moderately correlated (ρ = 0.28–0.46). The PCBs, trans-nonachlor, oxychlordane, mercury, and lead were also moderately correlated with each other (ρ = 0.14–0.44) (Figure 1).

**Table 2.** Distribution of chemicals during the first trimester of pregnancy, the MIREC study, Canada, 2008–2011 (n = 601)

Biomarker name	n	%>LOD	GM (GSD)	Percentiles				CHMS GM^a^
				25th	50th	75th	95th	
Metals (whole blood, μg/l)								
Arsenic	590	96.1	0.8 (2.0)	0.6	0.8	1.2	2.3	0.88
Cadmium	590	97.1	0.2 (2.1)	0.1	0.2	0.3	0.7	0.33
Lead	590	100.0	6.2 (1.6)	4.4	6.0	8.3	14	8.5
Mercury	590	90.5	0.6 (2.8)	0.3	0.7	1.3	2.8	0.66
Organochlorine pesticides (plasma, ng/g lipid)								
β-HCH	576	63.7	2.2 (2.7)	1.2	2.1	3.4	9.2	4.83
DDE	576	99.3	55 (2.1)	35	49	77	217	102
Oxychlordane	575	92.5	2.0 (1.7)	1.5	2.1	3.0	4.5	2.31
trans-Nonachlor	576	84.9	3.0 (1.8)	2.0	3.0	4.2	7.9	3.07
Per- and polyfluoroalkyl substances (plasma, μg/l)								
PFHxS	589	96.1	1.0 (2.2)	0.7	1.0	1.6	3.9	0.86
PFOS	589	99.8	4.3 (1.7)	3.1	4.4	6.2	9.9	4.4
PFOA	589	99.8	1.7 (1.8)	1.1	1.7	2.5	3.9	1.5
Polychlorinated biphenyls (plasma, ng/g lipid)								
PCB118	588	76.9	2.5 (1.8)	1.7	2.5	3.4	6.5	3.09
PCB138	588	94.4	4.5 (1.9)	2.9	4.3	6.4	14.5	5.46
PCB153	588	99.8	7.8 (2.0)	4.9	7.6	11.6	25.7	8.22
PCB180	588	96.4	5.4 (2.1)	3.2	5.2	8.2	20	5.79
Polybrominated diphenyl ethers (plasma, ng/g lipid)								
BDE47	583	66.0	7.6 (2.6)	4.1	7.0	12.2	39	10.83

a Concentration in Canadian women aged 20–39 years from of Canadian Health Measures Survey (CHMS). Cycle 1 (2007–2009) was used for arsenic, organochlorine pesticides, polychlorinated biphenyls, and polybrominated diphenyl ethers. Cycle 2 (2009–2011) was used for the cadmium, lead, mercury, and per- and polyfluoroalkyl substances.

BDE indicates brominated diphenyl ether; CHMS, Canadian Health Measures Survey; DDE, dichlorodiphenyldichloroethylene; GM, geometric mean; GSD, geometric standard deviation; LOD, limit of detection; MIREC, Maternal-Infant Research on Environmental Chemicals study; PCB, polychlorinated biphenyl; PFHxS, Perfluorohexanesulfonic acid; PFOA, Perfluorooctanoic acid; PFOS, Perfluorooctanesulfonic acid; β-HCH, β-hexachlorocyclohexane.

### Individual chemical-social responsiveness scale-2 associations

Lead, β-hexachlorocyclohexane (β-HCH), dichlorodiphenyldichloroethylene (DDE), PCB138, PCB153, PCB180, and brominated diphenyl ether-47 (BDE47) were associated with increased SRS-2 T-scores. Perfluorooctanoic acid (PFOA) and perfluorooctanesulfonic acid (PFOS) were associated with decreased SRS-2 T-scores (Table 3).

**Table 3.** Adjusted^a^ associations between chemical mixtures and SRS-2 T-scores using quantile g-computation, and individual chemicals and SRS-2 scores using linear regression, the MIREC study, Canada, 2008–2011 (n = 601)

Mixture or chemical name	Effect estimate (95% CI)^a^
Metals^b^	0.4 (−0.4, 1.2)
OC pesticides^c^	0.0 (−0.6, 0.7)
PFAS^d^	−0.5 (−1.1, 0.1)
PCBs^e^	0.4 (−0.2, 0.9)
All POPs^f^	0.1 (−0.9, 1.0)
Arsenic	0.2 (−0.4, 0.7)
Cadmium	0.2 (−0.3, 0.7)
Lead	0.9 (0.2, 1.6)
Mercury	−0.1 (−0.4, 0.3)
β-HCH	0.4 (0.0, 0.7)
DDE	0.3 (−0.1, 0.8)
Oxychlordane	0.0 (−0.7, 0.6)
trans-nonachlor	−0.3 (−1.0, 0.4)
PFHxS	0.0 (−0.5, 0.4)
PFOS	−0.4 (−1.1, 0.2)
PFOA	−0.6 (−1.2, 0.0)
PCB118	0.3 (−0.3, 0.9)
PCB138	0.7 (0.1, 1.2)
PCB153	0.5 (0.0, 1.1)
PCB180	0.4 (−0.1, 0.9)
BDE47	0.3 (−0.1, 0.6)

a Controls for the following variables: child sex, gestational folic acid supplementation, child age at SRS-2 assessment, HOME score, household income, relationship status, maternal education, maternal race, maternal age, parity, smoking status, city of residence, and year of enrollment. Effect estimates are pooled across 10 multiply imputed datasets. Stabilized inverse probability weights are applied.

b The metals mixture includes arsenic, cadmium, lead, and mercury.

c The OC pesticides mixture includes β-HCH, DDE, oxychlordane, trans-nonachlor.

d The PFAS mixture includes PFHxS, PFOS, and PFOA.

e The PCBs mixture includes PCB118, PCB138, PCB153, and PCB180.

f The POP mixture includes β-HCH, DDE, oxychlordane, trans-nonachlor, PFHxS, PFOS, PFOA, PCB118, PCB138, PCB153, PCB180, and BDE47.

BDE indicates brominated diphenyl ether; CI, confidence interval; DDE, dichlorodiphenyldichloroethylene; HOME, home observation for measurement of the environment; MICE, multiple imputation by chained equations; MIREC, Maternal-Infant Research on Environmental Chemicals study; OC, organochlorine; PCB, polychlorinated biphenyl; PFAS, Per- and polyfluoroalkyl substances; PFHxS, Perfluorohexanesulfonic acid; PFOA, Perfluorooctanoic acid; PFOS, Perfluorooctanesulfonic acid; POP, persistent organic pollutant; SRS-2, social responsiveness scale-2; β-HCH, β-hexachlorocyclohexane.

### Chemical mixture-social responsiveness scale-2 associations with quantile g-computation

The metal (Ψ = 0.4; 95% confidence interval [CI] = −0.4, 1.2) and PCB (Ψ = 0.4; 95% CI = −0.2, 0.9) mixtures were associated with an imprecise increase in SRS-2 T-scores. The PFAS mixture was associated with an imprecise decrease in SRS-2 T-scores (Ψ = −0.5; 95% CI = −1.1, 0.1). We observed null associations between the organochlorine pesticide and POP mixtures and SRS-2 T-scores (Table 3, Table S2; https://links.lww.com/EE/A353). The following chemicals had positive weights, suggesting a positive association with SRS-2 T-scores: cadmium, lead, DDE, perfluorohexanesulfonic acid, PCB138, PCB153, and BDE47. The following chemicals had negative weights, suggesting an inverse association with SRS-2 T-scores: mercury, β-HCH, oxychlordane, trans-nonachlor, PFOS, PFOA, PCB118, and PCB180. (Table S2; https://links.lww.com/EE/A353).

### Chemical mixture-social responsiveness scale-2 associations modified by folate and child sex

**Figure 2.**
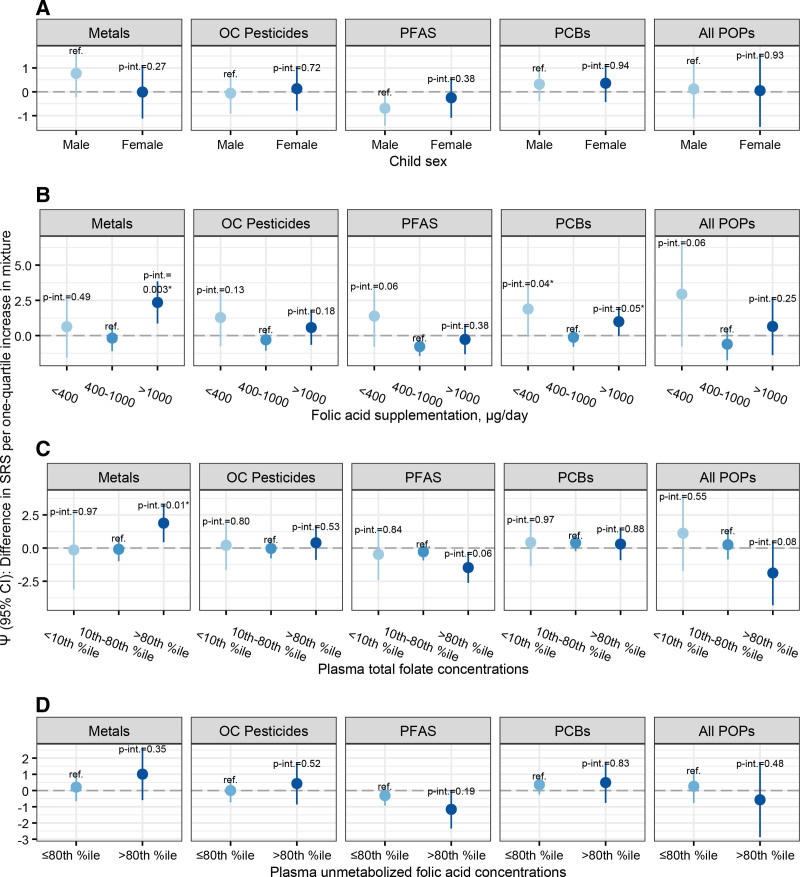
Adjusted associations between chemical mixtures and child SRS-2 T-scores using quantile g-computation and assessing interaction by child sex, gestational folic acid supplementation, plasma total folate concentrations, and unmetabolized folic acid concentrations, the MIREC study, Canada, 2008–2011 (n = 601). Controls for the following variables: child sex, child age at SRS-2 assessment, HOME score, household income, relationship status, maternal education, maternal race, maternal age, parity, smoking status, city of residence, and year of enrollment. Plot A additionally controls for folic acid supplementation. Effect estimates are pooled across 10 multiply imputed datasets. Stabilized inverse probability weights are applied. The metal mixture includes arsenic, cadmium, lead, and mercury. The OC pesticides mixture includes β-HCH, DDE, oxychlordane, and trans-nonachlor. The PFAS mixture includes PFHxS, PFOS, and PFOA. The PCB mixture includes PCB118, PCB138, PCB153, and PCB180. The POP mixture includes β-HCH, DDE, oxychlordane, trans-nonachlor, PFHxS, PFOS, PFOA, PCB118, PCB138, PCB153, PCB180, and BDE47. A, Mixture versus SRS-2 associations by child sex. B, Mixture versus SRS-2 associations by folic acid supplementation. C, Mixture versus SRS-2 associations by plasma total folate concentration. D, Mixture versus SRS-2 associations by plasma unmetabolized folic acid concentrations. *Denotes an interaction *P*-value (i.e., the *P*-value of the interaction term) less than 0.05. All *P*-values are two-sided. %ile indicates percentile; BDE, brominated diphenyl ether; DDE, dichlorodiphenyldichloroethylene; HOME, home observation for measurement of the environment; MIREC, Maternal-Infant Research on Environmental Chemicals study; PCB, polychlorinated biphenyl; PFHxS, Perfluorohexanesulfonic acid; PFOA, Perfluorooctanoic acid; PFOS, Perfluorooctanesulfonic acid; p-int, P-interaction; SRS-2, social responsiveness scale-2; UMFA, unmetabolized folic acid; β-HCH, β-hexachlorocyclohexane.

In addition to assessing the overall associations of these five mixtures, we assessed the mixture-SRS-2 associations among participants who were in the lowest, middle, and highest categories of FA supplementation and plasma total folate concentrations, and among male and female children. We found no clear evidence that child sex modified the mixture-SRS-2 associations (Figure 2A, Table S3; https://links.lww.com/EE/A353).

The associations between the metal mixture and SRS-2 T-scores were similar for participants in the lowest (<400 μg/d) (Ψ = 0.6; 95% CI = −1.6, 2.8) and middle (400–1,000 μg/d) (Ψ = −0.2; 95% CI = −1.1, 0.7) categories of FA supplementation (p-interaction = 0.49). Again, metal-SRS-2 associations were similar when comparing the lowest (<10th percentile) (Ψ = −0.1; 95% CI = −3.1, 2.8) and middle (10th–80th percentile) (Ψ = −0.1; 95% CI = −1.0, 0.8) categories of plasma total folate concentrations (p-interaction = 0.97). In contrast, the associations between the metal mixture and SRS-2 T-scores were stronger in the positive direction among participants in the highest (>1,000 μg/d) (Ψ = 2.4; 95% CI = 0.8, 3.9) versus middle (Ψ = −0.2; 95% CI = −1.1, 0.7) categories of FA supplementation (p-interaction = 0.003). We saw consistent results when comparing the highest (>80th percentile) (Ψ = 1.9; 95% CI = 0.4, 3.3) versus middle (Ψ = −0.1; 95% CI = −1.0, 0.8) categories of plasma total folate concentration (p-interaction = 0.01) (Table 4, Figure 2B and C, Tables S4 and S5; https://links.lww.com/EE/A353).

**Table 4.** Adjusted^a^ associations between chemical mixtures and SRS-2 T-scores using quantile g-computation, and individual chemicals and SRS-2 scores using linear regression, assessing modification by gestational folic acid supplementation and plasma total folate concentrations, the MIREC study, Canada, 2008–2011 (n = 601)

	Folic acid supplementation^b^ (µg/d)						Plasma total folate concentration^c^					
	Effect estimate (95% CI)^a^			p-interaction			Effect estimate (95% CI)^a^			p-interaction		
Mixture or chemical name	<400	400–1,000	>1,000	<400	400–1,000	>1,000	<10th %ile	10th–80th %ile	>80th %ile	<10th %ile	10th–80th %ile	>80th %ile
Metals^d^	0.6 (−1.6, 2.8)	−0.2 (−1.1, 0.7)	2.4 (0.8, 3.9)	0.49	ref.	0.003^i^	−0.1 (−3.1, 2.8)	−0.1 (−1.0, 0.8)	1.9 (0.4, 3.3)	0.97	ref.	0.01^i^
OC pesticides^e^	1.3 (−0.8, 3.3)	−0.3 (−1.1, 0.5)	0.6 (−0.7, 1.8)	0.13	ref.	0.18	0.2 (−1.7, 2.1)	0.0 (−0.8, 0.7)	0.4 (−0.9, 1.7)	0.80	ref.	0.53
PFAS^f^	1.4 (−0.8, 3.6)	−0.8 (−1.4, −0.1)	−0.3 (−1.3, 0.8)	0.06	ref.	0.38	−0.5 (−2.4, 1.5)	−0.3 (−0.9, 0.4)	−1.5 (−2.6, −0.3)	0.84	ref.	0.06
PCBs^g^	1.9 (0.0, 3.8)	−0.1 (−0.8, 0.6)	1.0 (0.0, 2.0)	0.04^i^	ref.	0.05^i^	0.4 (−1.4, 2.2)	0.4 (−0.2, 1.0)	0.3 (−0.9, 1.5)	0.97	ref.	0.88
All POPs^h^	2.9 (−0.8, 6.6)	−0.6 (−1.8, 0.5)	0.7 (−1.4, 2.7)	0.06	ref.	0.25	1.1 (−1.7, 4.0)	0.2 (−0.9, 1.4)	−1.9 (−4.3, 0.6)	0.55	ref.	0.08
Arsenic	−0.4 (−2.4, 1.6)	−0.4 (−1.1, 0.3)	1.2 (0.3, 2.1)	0.98	ref.	0.004^i^	0.7 (−1.6, 2.9)	−0.2 (−0.8, 0.5)	0.8 (−0.1, 1.7)	0.46	ref.	0.08
Cadmium	−0.3 (−2.1, 1.5)	0.0 (−0.6, 0.6)	0.7 (−0.1, 1.5)	0.72	ref.	0.12	−0.1 (−1.4, 1.3)	−0.3 (−0.9, 0.3)	1.2 (0.4, 2.0)	0.77	ref.	0.002^i^
Lead	2.3 (−0.6, 5.1)	0.3 (−0.5, 1.2)	2.2 (0.8, 3.5)	0.20	ref.	0.02^i^	3.8 (1.4, 6.3)	0.4 (−0.5, 1.2)	1.6 (0.2, 2.9)	0.008^i^	ref.	0.13
Mercury	−0.1 (−1.2, 0.9)	−0.4 (−0.8, 0.0)	0.8 (0.1, 1.4)	0.66	ref.	0.002^i^	−0.4 (−1.7, 0.9)	−0.2 (−0.6, 0.2)	0.4 (−0.3, 1.1)	0.77	ref.	0.13
β-HCH	0.3 (−0.8, 1.4)	0.0 (−0.4, 0.5)	0.9 (0.3, 1.5)	0.61	ref.	0.009^i^	0.1 (−1.1, 1.3)	0.4 (0.0, 0.8)	0.4 (−0.3, 1.0)	0.60	ref.	0.88
DDE	2.1 (0.5, 3.8)	−0.1 (−0.6, 0.5)	0.8 (−0.1, 1.7)	0.01^i^	ref.	0.07	1.0 (−0.4, 2.3)	0.3 (−0.2, 0.9)	−0.1 (−1.3, 1.0)	0.36	ref.	0.41
Oxychlordane	0.7 (−1.7, 3.2)	−0.4 (−1.2, 0.5)	0.5 (−0.7, 1.7)	0.39	ref.	0.21	−1.0 (−3.2, 1.3)	−0.2 (−0.9, 0.6)	0.7 (−0.7, 2.2)	0.48	ref.	0.24
trans-Nonachlor	0.7 (−1.5, 2.9)	−0.4 (−1.3, 0.5)	−0.3 (−1.7, 1.0)	0.32	ref.	0.96	−0.5 (−2.5, 1.4)	−0.3 (−1.3, 0.6)	0.0 (−1.6, 1.5)	0.86	ref.	0.68
PFHxS	0.5 (−1.6, 2.7)	−0.2 (−0.7, 0.2)	0.4 (−0.4, 1.2)	0.49	ref.	0.14	−0.7 (−2.5, 1.0)	0.0 (−0.4, 0.5)	−0.3 (−1.3, 0.7)	0.38	ref.	0.53
PFOS	3.3 (0.8, 5.8)	−1.0 (−1.7, −0.3)	0.3 (−0.9, 1.4)	0.001^i^	ref.	0.07	1.1 (−0.8, 3.1)	−0.4 (−1.2, 0.3)	−1.2 (−2.4, 0.1)	0.13	ref.	0.27
PFOA	−0.4 (−2.9, 2.2)	−0.8 (−1.5, −0.1)	−0.1 (−1.2, 1.0)	0.74	ref.	0.28	−0.8 (−3.0, 1.4)	−0.4 (−1.1, 0.3)	−1.1 (−2.4, 0.2)	0.74	ref.	0.32
PCB118	1.5 (−0.3, 3.2)	−0.3 (−1.0, 0.4)	1.5 (0.4, 2.6)	0.06	ref.	0.006^i^	−0.3 (−2.2, 1.6)	0.5 (−0.2, 1.1)	0.3 (−1.0, 1.6)	0.45	ref.	0.79
PCB138	2.2 (0.5, 4.0)	0.1 (−0.5, 0.8)	1.5 (0.5, 2.6)	0.03^i^	ref.	0.02^i^	0.9 (−0.8, 2.6)	0.7 (0.1, 1.4)	0.4 (−0.7, 1.6)	0.83	ref.	0.63
PCB153	1.9 (0.3, 3.5)	0.1 (−0.5, 0.7)	1.3 (0.2, 2.3)	0.04^i^	ref.	0.05	0.6 (−1.1, 2.3)	0.6 (0.0, 1.2)	0.4 (−0.6, 1.5)	>0.99	ref.	0.79
PCB180	1.8 (0.2, 3.3)	0.1 (−0.5, 0.6)	0.9 (−0.1, 1.8)	0.04^i^	ref.	0.13	0.3 (−1.3, 1.9)	0.4 (−0.1, 1.0)	0.3 (−0.6, 1.2)	0.91	ref.	0.79
BDE47	1.9 (0.1, 3.7)	0.1 (−0.3, 0.5)	0.4 (−0.2, 0.9)	0.06	ref.	0.39	1.1 (−0.1, 2.4)	0.5 (0.1, 0.9)	−0.5 (−1.2, 0.2)	0.31	ref.	0.01^i^

a Controls for the following variables: child sex, child age at SRS-2 assessment, HOME score, household income, relationship status, maternal education, maternal race, maternal age, parity, smoking status, city of residence, and year of enrollment. Effect estimates are pooled across 10 multiply imputed datasets. Stabilized inverse probability weights are applied.

b Folic acid supplementation was primarily measured via a survey conducted at 16 weeks gestation, which queried intake in the past 30 days. We also used data from the 24-hour recall version of this survey and a questionnaire completed at study enrollment (6–13 weeks gestation).

c The sum of 5-formyl-THF, 5-10-methylene-THF, THF, UMFA, and 5-methyl-THF.

d The metal mixture includes arsenic, cadmium, lead, and mercury.

e The OC pesticides mixture includes β-HCH, DDE, oxychlordane, and trans-Nonachlor.

f The PFAS mixture includes PFHxS, PFOS, and PFOA.

g The PCBs mixture includes PCB118, PCB138, PCB153, and PCB180.

h The POP mixture includes β-HCH, DDE, oxychlordane, trans-nonachlor, PFHxS, PFOS, PFOA, PCB118, PCB138, PCB153, PCB180, and BDE47.

i Denotes an interaction *P*-value (i.e., the *P*-value of the interaction term) less than 0.05. All *P*-values are two-sided.

%ile indicates percentile; BDE, brominated diphenyl ether; CI, confidence interval; DDE, dichlorodiphenyldichloroethylene; HOME, home observation for measurement of the environment; MICE, multiple imputation by chained equations; MIREC, Maternal-Infant Research on Environmental Chemicals study; OC, organochlorine; PCB, polychlorinated biphenyl; PFAS, per- and polyfluoroalkyl substances; PFHxS, Perfluorohexanesulfonic acid; PFOA, Perfluorooctanoic acid; PFOS, Perfluorooctanesulfonic acid; POP, persistent organic pollutant; Ref, reference level; SRS-2, social responsiveness scale-2; THF, tetrahydrofolate; UMFA, unmetabolized folic acid; β-HCH, β-hexachlorocyclohexane.

The associations between the organochlorine pesticide mixture and SRS-2 T-scores were null among participants in the middle category of FA supplementation (Ψ = −0.3; 95% CI = −1.1, 0.5). These associations were slightly stronger in the positive direction among participants in the lowest (Ψ = 1.3; 95% CI = −0.8, 3.3) and highest (Ψ = 0.6; 95% CI = −0.7, 1.8) categories of FA supplementation (p-interaction_lowest versus middle_ = 0.13, interaction_highest versus middle_ = 0.18). We found no evidence that the organochlorine pesticide-SRS-2 associations were modified by plasma total folate concentrations (Table 4, Figure 2B and C, Tables S4 and S5; https://links.lww.com/EE/A353).

While the PFAS-SRS-2 associations were inverse among participants in the middle category of FA supplementation (Ψ = −0.8; 95% CI = −1.4, −0.1), association were positive among participants in the lowest category of FA supplementation (Ψ = 1.4; 95% CI = −0.8, 3.6) (p-interaction = 0.06). The PFAS-SRS-2 associations were similar among participants in the highest and middle categories of FA supplementation (p-interaction = 0.38). The results were not consistent when assessing modification by plasma total folate concentrations. PFAS-SRS-2 associations were similar in the lowest and middle categories of plasma total folate concentrations (p-interaction = 0.84). PFAS-SRS-2 associations were stronger in the inverse direction among participants in the highest (Ψ = −1.5; 95% CI = −2.6, −0.3) versus middle (Ψ = −0.3; 95% CI = −0.9, 0.4) categories of plasma total folate concentrations (p-interaction = 0.06) (Table 4, Figure 2B and C, Tables S4 and S5; https://links.lww.com/EE/A353).

Next, there was a “u-shaped” trend where the PCB-SRS-2 associations were stronger in the positive direction among participants in the lowest (Ψ = 1.9; 95% CI = 0.0, 3.8) and highest (Ψ = 1.0; 95% CI = 0.0, 2.0) categories of FA supplementation compared with the middle category (Ψ = −0.1; 95% CI = −0.8, 0.6) (p-interaction_lowest versus middle_ = 0.04, interaction_highest versus middle_ = 0.05). There was no evidence that plasma total folate concentrations modified the PCB-SRS-2 associations (Table 4, Figure 2B and C, Tables S4 and S5; https://links.lww.com/EE/A353).

The POP-SRS-2 associations were stronger in the positive direction among participants in the lowest (Ψ = 2.9; 95% CI = −0.8, 6.6) versus middle (Ψ = −0.6; 95% CI = −1.8, 0.5) categories of FA supplementation (p-interaction = 0.06). The POP-SRS-2 associations were slightly stronger in the positive direction among participants in the highest (Ψ = 0.7; 95% CI = −1.4, 2.7) versus middle (Ψ = −0.6; 95% CI = −1.8, 0.5) categories of FA supplementation (p-interaction = 0.25). Next, the POP-SRS-2 associations were similar for participants in the lowest and middle categories of plasma total folate concentrations (p-interaction = 0.55). But these associations were stronger in the inverse direction among participants in the highest (Ψ = −1.9; 95% CI = −4.3, 0.6) versus middle (Ψ = 0.2; 95% CI = −0.9, 1.4) categories of plasma total folate concentrations (p-interaction = 0.08) (Table 4, Figure 2B and C, Tables S4 and S5; https://links.lww.com/EE/A353).

### Exploratory and sensitivity analyses

To complement our quantile g-computation analysis, we performed an exploratory analysis with weighted quantile sum regression. The results were largely consistent with our main analysis. However, the PFAS-SRS-2 associations were stronger in the inverse direction when we used weighted quantile sum regression ($\beta_{\left(-\right)}$ = −1.0; 95% CI = −1.7, −0.3) versus quantile g-computation (Ψ = −0.5; 95% CI = −1.1, 0.1). The PCB-SRS-2 associations were stronger in the positive direction when we used weighted quantile sum regression ($\beta_{\left(+\right)}$ = 0.8; 95% CI = 0.1, 1.4) versus quantile g-computation (Ψ = 0.4; 95% CI = −0.2, 0.9) (Table 3, Table S2; https://links.lww.com/EE/A353, Table S6; https://links.lww.com/EE/A353).

Next, we assessed whether individual chemicals were modified by FA supplementation and plasma total folate concentrations. There was evidence that the associations between all metals (arsenic, cadmium, lead, and mercury) and SRS-2 T-scores were stronger in the positive direction in among participants in the highest versus middle categories of both folate exposure variables. But it was less clear whether FA supplementation modified arsenic-SRS-2 associations and whether plasma total folate concentrations modified mercury-SRS-2 associations. The lead-SRS-2 associations were stronger in the positive direction among participants in the lowest versus middle categories of both folate exposure variables (Table 4). The DDE, PFOS, PCB118, PCB138, and PCB153 versus SRS-2 associations were ‘u-shaped’, or stronger in the positive direction in the lowest and highest categories of FA supplementation compared with the middle category. The PCB180 and BDE47 versus SRS-2 associations were stronger in the positive direction among participants in the lowest versus middle categories of FA supplementation. The β-HCH-SRS-2 associations were stronger in the positive direction among participants in the highest versus middle categories of FA supplementation. However, there was less evidence that plasma total folate concentrations modified the associations between individual POPs and SRS-2 scores. The PFOS-SRS-2 associations were stronger in the positive direction among participants in the lowest versus middle categories of plasma total folate concentrations. In contrast, the BDE47-SRS-2 associations were stronger in the inverse direction among participants in the highest versus middle categories of plasma total folate concentrations (Table 4).

The metal-SRS-2 associations were similar among participants in the higher (>80th percentile) (Ψ = 1.0; 95% CI = −0.6, 2.6) versus lowest (≤80th percentile) (Ψ = 0.2; 95% CI = −0.7, 1.1) category of unmetabolized FA concentrations (p-interaction = 0.35) (Figure 2D, Table S7; https://links.lww.com/EE/A353). The difference in associations was clearer when comparing participants in the highest (Ψ = 2.4; 95% CI = 0.8, 3.9) versus middle (Ψ = −0.2; 95% CI = −1.1, 0.7) categories of FA supplementation (p-interaction = 0.003) (Table 4, Figure 2B, Table S3; https://links.lww.com/EE/A353). Additionally, controlling for fish consumption during pregnancy did not change our results (Table S8; https://links.lww.com/EE/A353). To probe the direction of selection bias, we compared our results with and without IPW. After applying IPW, the PFAS- and POP-SRS-2 associations moved towards the null, and the metal- and PCB-SRS-2 associations move away from the null (Table 3, Table S9; https://links.lww.com/EE/A353). The evidence that gestational folate exposure modified the mixture-SRS-2 associations was weaker when IPW was not used (Table 4, Figure S3; https://links.lww.com/EE/A353, Tables S10 and S11; https://links.lww.com/EE/A353).

## Discussion

We examined the associations between five mixtures of gestational environmental chemicals and childhood autistic-like behaviors in a Canadian pregnancy and birth cohort. The PFAS mixture was associated with an imprecise decrease in SRS-2 T-scores. The metal and PCB mixtures were associated with an imprecise increase in SRS-2 T-Scores. These findings were robust when additionally controlling for gestational fish consumption. In some cases, these findings were stronger when assessing individual chemicals and when using a complementary mixture method (weighted quantile sum regression). This article is the first, to our knowledge, to assess whether the associations between chemical mixtures and autistic-like behaviors or autism diagnosis could be modified by folate. The metal mixture was most strongly associated with increased SRS-2 T-scores among participants who had high (>1,000 μg/d) FA supplementation or were in the highest category (>80th percentile) of plasma total folate concentrations. The associations between the PCB mixture and SRS-2 T-scores were stronger among participants who high versus adequate (400–1,000 μg/d) FA supplementation, but this was not observed with plasma total folate concentrations. The PFAS, PCB, and POP mixtures were more associated with increased SRS-2 T-scores among participants who had low (<400 μg/d) versus adequate FA supplementation, but this trend was not observed when we assessed modification by plasma total folate concentrations. Finally, we found no clear evidence that child sex modified the mixture-SRS-2 associations.

Our analysis of the associations between the prenatal chemical mixtures and SRS-2 T-scores adds to the nuanced body of research on this topic. We observed a small positive but imprecise association between the metal mixture and SRS-2 T-scores. A previous study also found no association between a mixture of prenatal metals and autism diagnosis.^5^ Still, previous research tends to suggest that prenatal exposure to cadmium,^5,6^ and lead,^5–8^ are positively associated with autism or autistic-like behaviors. Findings are less clear for arsenic,^5–7^ and mercury.^5–7^ We identified null associations between the organochlorine pesticide mixture and SRS-2 T-scores. Previous research on the associations between individual organochlorine pesticide congeners and outcomes is mixed, with inconsistent findings for DDE^10–14^ and trans-nonachlor,^11,12^ inverse associations for β-HCH,^11^ and null associations for oxychlordane.^11^ Next, we observed inverse, borderline-significant associations between the PFAS mixture and SRS-2 T-scores, consistent with Skogheim et al.^17^ Previous research on the associations between PFAS and autism outcomes is mixed,^15–20^ but tends to suggest inverse associations for PFOA and PFOS.^15–17^ We observed an imprecise positive relationship between the PCB mixture and SRS-2 T-scores, but findings were stronger for PCB138 and PCB153. The associations between individual PCB congeners and autism outcomes are mixed, with inconsistent findings for individual congeners.^10–14^ Finally, we observed null associations between the POP mixture and SRS-2 T-scores. Two previous studies also found unclear associations between a mixture of various POPs and autism outcomes.^13,86^ These previous studies may differ from ours with respect to population, design, or chemical concentration distributions. Previous “mixture” studies used different statistical methodologies and/ or had mixtures that were composed of different chemicals than our study.^5,13,17,86^

Next, we examined whether the chemical-SRS-2 associations could be modified by two indicators of gestational folate exposure (FA supplementation and plasma total folate concentrations). As explained in the introduction, folate and environmental chemicals may have antagonistic properties.^43^ We therefore hypothesized that the chemical-SRS-2 associations would be stronger when gestational folate exposure was lower. Our results suggest that the PFAS, PCB, and POP mixtures (and to a lesser extent, the organochlorine pesticide mixture) were more strongly associated with increased SRS-2 T-scores among participants with low FA supplementation compared with those with adequate supplementation. However, this finding was not replicated when comparing participants in the lowest versus middle categories of plasma total folate concentrations. Next, the associations between the PFAS and POP mixtures were stronger in the inverse direction among participants in the highest (>80th percentile) versus middle (10th–80th percentile) categories of plasma total folate concentrations. This was not observed when assessing modification by FA supplementation. The inconsistent findings between these two indicators of folate exposure diminishes our confidence in our results, as we expected both indicators of folate exposure to modify mixture-SRS-2 associations in a similar manner. Still, these indicators are not the same and have their own limitations (as described below), so some inconsistencies are plausible. Our results contrast with a previous paper that found no evidence that prenatal vitamin intake modified the associations between individual PFAS congeners and autism diagnosis.^44^ These inconsistent findings may be because this article defined “FA supplementation” differently than us, did not directly assess gestational PFAS concentrations, and did not use IPW.^44^

Next, the associations between the metal mixture and SRS-2 T-scores were stronger among participants in the highest category of FA supplementation and plasma total folate concentrations compared with the middle categories. The PCB mixture, as well as β-HCH, DDE, and PFOS were more strongly associated with increased SRS-2 T-scores among participants with high versus adequate FA supplementation, but not high versus middle plasma total folate concentrations. To investigate this further, we assessed modification by unmetabolized FA concentrations. Higher FA supplementation and plasma total folate concentrations are associated with higher plasma-unmetabolized FA concentrations.^74^ Previous research in mice and tissue samples suggests that elevated unmetabolized FA concentrations may interfere with the dihydrofolate reductase and methylenetetrahydrofolate reductase enzymes, key enzymes involved in folate-mediated one-carbon metabolism.^87–89^ Unmetabolized FA concentrations might explain why the mixture-SRS-2 associations were stronger among participants with higher folate exposure. If this were the case, mixture-SRS-2 associations should be especially strong among participants with high unmetabolized FA concentrations, but this was not observed. The metal-SRS-2 associations, for example, were slightly stronger among participants in the high (>80th percentile) versus low (≤80th percentile) categories of unmetabolized FA concentrations. But the contrast was sharper when comparing the high to middle categories of FA supplementation and plasma total folate concentrations. This suggests that a mechanism other than unmetabolized FA may explain why some of the mixture-SRS-2 associations were stronger among participants with higher folate exposure. Further research replicating this result and probing a mechanism may be needed.

There are numerous limitations associated with the two indicators of gestational folate exposure we utilized. FA supplementation does not consider dietary folate intake (from products with naturally occurring folate or fortified with FA). To reduce missing data, we assessed FA supplementation at three different time points and assumed that supplementation was consistent throughout, which may result in misclassification.^42^ FA supplementation is self-reported, making it susceptible to recall and social desirability bias.^90^. In contrast, plasma total folate concentrations are a more direct indicator of folate exposure, making it less susceptible to bias. However, plasma total folate concentrations may be more prone to measurement error because participants were not required to fast before providing samples.^53,74,75^ We categorized plasma total folate concentrations using fixed cutoffs that may not be clinically or biologically relevant.^91^ Our use of fixed cutoffs instead of a more flexible approach (i.e., splines) may have prevented us from seeing stronger evidence of modification. Finally, over 99% of participants had plasma total folate concentrations >25.5 nmol/l, a level associated with maximal neural tube defect risk reduction among vitamin B12 adequate individuals^92^ (which is the case in the MIREC cohort^74^). Unlike other jurisdictions, the fortification of FA into grain products is mandatory in Canada.^93,94^ There may be stronger evidence of modification in a sample with lower folate exposure.

This article has several additional limitations. Our results may be less generalizable because MIREC participants were not representative of the Canadian population or other countries. MIREC participants were older, more affluent, and were more likely to identify as White.^48,49^ Geometric mean concentrations of most chemicals were lower in our sample than in 20–39 year-old nonpregnant Canadian women who participated in cycle 1 (2007–2009) or cycle 2 (2009–2011) of the Canadian Health Measurement Survey.^95,96^ Next, we used the preschool-aged version of the SRS-2, which may underestimate autistic-like behaviors relative to the school-aged version.^97^ Chemical mixtures were based on established chemical classes to estimate the effect of eliminating exposure to that chemical class.^76,98^ However, the chemicals within a given mixture were sometimes dissimilar. The “metal” mixture included metals that may have different exposure sources.^27,99^ The “POP” mixture (which included chemicals that were classified as POPs by the Stockholm Convention^4^) included uncorrelated chemicals (namely BDE47 and PFAS chemicals).

We assessed the relationship between gestational mixtures of environmental chemicals autistic-like behaviors during childhood, and whether this relationship was modified by gestational folate exposure in a Canadian pregnancy and birth cohort. Our results suggest an inverse relationship between a gestational mixture of PFAS chemicals and autistic-like behaviors. Our results also suggest that the metal mixture was most strongly associated with increased autistic-like behaviors among participants with higher FA supplementation or plasma total folate concentrations. The PCB mixture was more strongly associated with increased autistic-like behaviors among participants with lower and higher FA supplementation, but this trend was not replicated when assessing modification by plasma total folate concentrations. The PFAS and POP mixtures were most strongly associated with more autistic-like behaviors among participants with low FA supplementation, but this trend was not observed among participants with lower plasma total folate concentrations. Further research is needed, particularly in populations with higher environmental chemical exposure and lower folate exposure.

## Conflicts of interest statement

The authors declare that they have no conflicts of interest with regard to the content of this report.

## Acknowledgements

We are grateful to all the participants who took part in the MIREC Study, as well as to all the study staff. We are also grateful to Michael Borghese and John Krzeczkowski for preparing the gestational fish consumption variable and advising how to use it.

## Supplementary Material
