# Supplementary material for: Association between gestational environmental chemical mixtures and folate exposures with autistic behaviors in a Canadian birth cohort
Source: Environ Epidemiol. 2025 Jun 11;9(4):e402. doi: 10.1097/EE9.0000000000000402 (PMC12160746; doi:10.1097/EE9.0000000000000402)
Supplement: Supplementary file 1 [file ee9-9-e402-s001.pdf]

Supplementary material for:

Association between gestational environmental chemical mixtures and folate exposures with autistic behaviors in a Canadian birth cohort

**Item**	**Page number**
Table of contents	1
Appendix 1: Further details on our use of multiple imputation by chained equations.	2
Table S1. Comparison of study and full sample's sociodemographic characteristics, MIREC study, Canada, 2008-11.	3
Table S2. Adjusted associations between chemical mixtures and SRS-2 T-scores using quantile g-computation, including weights, the MIREC study, Canada, 2008-2011 (n = 601).	5
Table S3. Adjusted associations between chemical mixtures and SRS-2 T-scores using quantile g-computation and **assessing modification by child sex**, including quantile g-computation weights, the MIREC study, Canada, 2008-2011 (n = 601).	6
Table S4. Adjusted associations between chemical mixtures and SRS-2 T-scores using quantile g-computation and **assessing modification by gestational folic acid supplementation**, including quantile g-computation weights, the MIREC study, Canada, 2008-2011 (n = 601).	7
Table S5. Adjusted associations between chemical mixtures and SRS-2 T-scores using quantile g-computation and **assessing modification by plasma total folate concentrations**, including quantile g-computation weights, the MIREC study, Canada, 2008-2011 (n = 601).	8
Table S6. Adjusted associations between chemical mixtures and SRS-2 T-scores **using weighted quantile sum regression**, including weighted quantile sum weights, the MIREC study, Canada, 2008-2011 (n = 601).	9
Table S7. Adjusted associations between chemical mixtures and SRS-2 T-scores using quantile g-computation and **assessing modification by plasma unmetabolized folic acid concentrations**, including quantile g-computation weights, the MIREC study, Canada, 2008-2011 (n = 601).	10
Table S8. Adjusted associations (**additionally controlling for gestational fish consumption**) between chemical mixtures and SRS-2 T-scores using quantile g-computation, including quantile g-computation weights, the MIREC study, Canada, 2008-2011 (n = 601).	11
Table S9. Adjusted associations between chemical mixtures and SRS-2 T-scores using quantile g-computation **without inverse probability weighting**, the MIREC study, Canada, 2008-2011 (n = 601).	12
Table S10. Adjusted associations between chemical mixtures and SRS-2 T-scores using quantile g-computation and assessing modification by gestational folic acid supplementation **without inverse probability weighting**, the MIREC study, Canada, 2008-2011 (n = 601).	13
Table S11. Adjusted associations between chemical mixtures and SRS-2 T-scores using quantile g-computation and assessing modification by plasma total folate concentrations **without inverse probability weighting**, the MIREC study, Canada, 2008-2011 (n = 601).	14
Figure S1. Flow chart of study participants in sample, the MIREC Study, Canada, 2008-11.	15
Figure S2. Directed acyclic graph depicting our assumptions about the relationship between gestational environmental chemical mixtures and autistic behaviors.	16
Figure S3. Adjusted associations between chemical mixtures and child SRS-2 T-scores using quantile g-computation and assessing interaction by gestational folic acid supplementation and plasma total folate concentrations, **without inverse probability weighting**, the MIREC study, Canada, 2008-2011	17
References	19

Appendix 1: Further details on our use of multiple imputation by chained equations.

In all regression analyses in this paper, we used multiple imputation by chained equations (MICE) to impute missing data.[1,2] Specifically, we imputed missing chemical concentrations, plasma total folate concentrations, unmetabolized folic acid concentrations, income, education, Home Observation for Measurement of the Environment (HOME) scores, and fish consumption. All regression coefficients presented in this study were pooled from the same 10 multiply imputed datasets.

However, we did not pool the quantile g-computation weights, and instead reported the weights from the first imputation. One of the key properties of quantile g-computation is that for a given mixture, the positive weights of all chemicals must sum to 1, and the negative weights must sum to -1.[3] But if a given chemical's weight in a given mixture does not have the same sign for all of the multiply imputed datasets (which was the case for all of the mixtures and analyses we considered, see Tables S3-5. S7-11), then the pooled weights will not have this property.

Unlike quantile g-computation, weighted quantile sum generates weights that have a value between 0 and 1.[4] Therefore, the sum of the pooled weights will always be 1. For this reason, we pooled the weights across the 10 multiply imputed datasets for the weighted quantile sum regression analysis (Figure S6).

Finally, we reiterate that MICE was not used to impute missing SRS-2 T-scores. We used inverse probability weighting to address missing SRS-2 T-scores, which are likely subject to non-random follow.[5]

Table S1. Comparison of study and full sample's sociodemographic characteristics, MIREC study, Canada, 2008-11.

Variable	Study sample (mother-child pairs with an SRS-2 score) n (%)	Full sample (singleton live births) without an SRS-2 score n (%)	p
All	601 (100.0)	1261 (100.0)	
Child sex			0.009*
Male	290 (48.3)	689 (54.6)	
Female	311 (51.7)	567 (45.0)	
Missing	0 (0.0)	5 (0.4)	
Maternal age at enrollment (years)			p < .001*
18 - 29	132 (22.0)	374 (29.7)	
30 - 35	292 (48.6)	493 (39.1)	
≥36	177 (29.5)	394 (31.2)	
Living with partner			0.04*
Yes	580 (96.5)	1187 (94.1)	
No	21 (3.5)	74 (5.9)	
Maternal race			p < .001*
White	539 (89.7)	1052 (83.4)	
Other	62 (10.3)	209 (16.6)	
Education level			p < .001*
High school or less	30 (5.0)	131 (10.4)	
College or trade school	167 (27.8)	371 (29.4)	
Undergraduate university degree	239 (39.8)	445 (35.3)	
Graduate university degree	163 (27.1)	314 (24.9)	
Missing	2 (0.3)	0 (0.0)	
Annual household income ($CAD)			0.09
≤$40,000	61 (10.1)	165 (13.1)	
$40,001 - $80,000	173 (28.8)	301 (23.9)	
$80,001 - $100,000	116 (19.3)	243 (19.3)	
>$100,000	231 (38.4)	486 (38.5)	
Missing	20 (3.3)	66 (5.2)	
Parity			0.63
Nulliparous	261 (43.4)	553 (43.9)	
Uniparous	251 (41.8)	503 (39.9)	
Multiparous	89 (14.8)	205 (16.3)	
HOME score			0.10
≥48 (median)	323 (53.7)	2 (0.2)	
<48 (median)	260 (43.3)	7 (0.6)	
Missing	18 (3.0)	1252 (99.3)	
Smoked during pregnancy			p < .001*
Yes[a]	47 (7.8)	174 (13.8)	
No	554 (92.2)	1086 (86.1)	
Missing	0 (0.0)	1 (0.1)	
Year of enrollment			p < .001*
2008	10 (1.7)	128 (10.2)	
2009	187 (31.1)	536 (42.5)	
2010	381 (63.4)	529 (42.0)	
2011	23 (3.8)	68 (5.4)	
First trimester fish consumption			0.53
0-2 times per month	220 (36.6)	497 (39.4)	
3-7 times per month	209 (34.8)	421 (33.4)	
≥8 times per month	169 (28.1)	340 (27.0)	
Missing	3 (0.5)	3 (0.2)	

Table S1. Comparison of study and full sample's sociodemographic characteristics, MIREC study, Canada, 2008-11.

Variable	Study sample (mother-child pairs with an SRS-2 score) n (%)	Full sample (singleton live births) without an SRS-2 score n (%)	p
Folic acid supplementation[b]			0.90
<400 µg per day	34 (5.7)	65 (5.2)	
400 - 1000 µg per day	423 (70.4)	890 (70.6)	
>1000 µg per day	144 (24.0)	306 (24.3)	
Plasma total folate concentrations[c]			0.27
<65.6 nmol/L	48 (8.0)	155 (12.3)	
≥65.6 nmol/L & ≤125 nmol/L	409 (68.1)	785 (62.3)	
>125 nmol/L	116 (19.3)	229 (18.2)	
Missing	28 (4.7)	92 (7.3)	
Unmetabolized folic acid concentrations[d]			0.77
≤13.6 nmol/L	461 (76.7)	927 (73.5)	
>13.6 nmol/L	112 (18.6)	242 (19.2)	
Missing	28 (4.7)	92 (7.3)	

*: Denotes a Chi-square (comparing the study and full sample) test P-value less than 0.05. All P-values are two-sided.

a: Includes current smokers and individuals who quit during pregnancy. 'Non-smoker' includes participants who did not smoke and former smokers.

b: Folic acid supplementation was primarily measured via a survey conducted at 16 weeks gestation, which queried intake in the past 30 days. We also used data from the 24-hour recall version of this survey and a questionnaire completed at study enrollment (6-13 weeks gestation).

c: Sum of 5-formyl-THF, 5-10-methylene-THF, THF, UMFA, 5-methyl-THF. 65.6 nmol/L and 125 nmol/L are the 10th and 80th percentiles of plasma total folate concentrations in the study sample.

d: 13.6 nmol/L is the 80th percentile of unmetabolized folic acid concentrations in the study sample.

Abbreviations: CAD, Canadian dollar; HOME, Home Observation for Measurement of the Environment; MIREC, Maternal-Infant Research on Environmental Chemicals Study; SD, Standard Deviation; SRS, Social Responsiveness Scale.

Table S2. Adjusted[a] associations between chemical mixtures and SRS-2 T-scores using quantile g-computation, including weights, the MIREC study, Canada, 2008-2011 (n = 601).

Mixture name	Metals	OC Pesticides	PFAS	PCBs	All POPs
Ψ (95% CI)[a]	0.4 (-0.4, 1.2)	0.0 (-0.6, 0.7)	-0.5 (-1.1, 0.1)	0.4 (-0.2, 0.9)	0.1 (-0.9, 1.0)
Biomarker names	Weights[b]				
Arsenic	-0.08				
Cadmium	0.25				
Lead	0.75				
Mercury	-0.92				
β-HCH		-0.32			-0.07
DDE		1			-0.05
Oxychlordane		-0.41			-0.08
trans-Nonachlor		-0.27			-0.15
PFHxS			1		0.23
PFOS			-0.7		-0.34
PFOA			-0.3		-0.15
PCB118				-0.47	-0.03
PCB138				0.46	0.27
PCB153				0.54	0.3
PCB180				-0.53	-0.12
BDE47					0.21

a: Controls for the following variables: child sex, gestational folic acid supplementation, child age at SRS-2 assessment, HOME score, household income, relationship status, maternal education, maternal race, maternal age, parity, smoking status, city of residence, and year of enrollment. Effect estimates are pooled across 10 multiply imputed datasets. Stabilized inverse probability weights are applied.

b: Only the weights from the first MICE imputation, not the pooled weights, are displayed.

Abbreviations: BDE, brominated diphenyl ether; CI; Confidence interval; DDE, Dichlorodiphenyldichloroethylene; HOME, Home Observation for Measurement of the Environment; MICE, Multiple imputation by chained equations; MIREC, Maternal-Infant Research on Environmental Chemicals Study; OC, Organochlorine; PCB, Polychlorinated biphenyl; PFAS, Per- and polyfluoroalkyl substances; PFHxS, Perfluorohexanesulfonic acid; PFOA, Perfluorooctanoic acid; PFOS, Perfluorooctanesulfonic acid; POP, Persistent organic pollutant; SRS-2, Social Responsiveness Scale-2; β-HCH, β-Hexachlorocyclohexane.

Table S3. Adjusted[a] associations between chemical mixtures and SRS-2 T-scores using quantile g-computation and **assessing modification by child sex**, including quantile g-computation weights, the MIREC study, Canada, 2008-2011 (n = 601).

Mixture name	Metals		OC Pesticides		PFAS		PCBs		All POPs	
Child sex	Male	Female	Male	Female	Male	Female	Male	Female	Male	Female
Ψ (95% CI)[a]	0.8 (-0.2, 1.8)	0.0 (-1.1, 1.1)	-0.1 (-0.9, 0.8)	0.1 (-0.8, 1.0)	-0.7 (-1.4, 0.0)	-0.2 (-1.1, 0.6)	0.3 (-0.4, 1.0)	0.4 (-0.4, 1.1)	0.1 (-1.1, 1.4)	0.0 (-1.5, 1.6)
Biomarker names	Weights[b]									
Arsenic	0.11	-0.23								
Cadmium	0.07	0.53								
Lead	0.78	0.47								
Mercury	0.04	-0.77								
β-HCH			-0.07	-0.25					-0.03	-0.11
DDE			1	-0.75					0.13	-0.34
Oxychlordane			-0.21	0.2					-0.06	0.06
trans-Nonachlor			-0.72	0.8					-0.38	0.23
PFHxS					1	1			0.14	0.2
PFOS					-0.68	-0.75			-0.19	-0.28
PFOA					-0.32	-0.25			-0.13	-0.05
PCB118							-0.26	-0.56	0.06	-0.1
PCB138							0.26	0.61	0.04	0.43
PCB153							0.74	-0.44	0.43	-0.12
PCB180							-0.74	0.39	-0.21	0.01
BDE47									0.21	0.08

a: Controls for the following variables: gestational folic acid supplementation, child age at SRS-2 assessment, HOME score, household income, relationship status, maternal education, maternal race, maternal age, parity, smoking status, city of residence, and year of enrollment. Effect estimates are pooled across 10 multiply imputed datasets. Stabilized inverse probability weights are applied.

b: Only the weights from the first MICE imputation, not the pooled weights, are displayed.

Abbreviations: BDE, brominated diphenyl ether; CI; Confidence interval; DDE, Dichlorodiphenyldichloroethylene; HOME, Home Observation for Measurement of the Environment; MICE, Multiple imputation by chained equations; MIREC, Maternal-Infant Research on Environmental Chemicals Study; OC, Organochlorine; PCB, Polychlorinated biphenyl; PFAS, Per- and polyfluoroalkyl substances; PFHxS, Perfluorohexanesulfonic acid; PFOA, Perfluorooctanoic acid; PFOS, Perfluorooctanesulfonic acid; POP, Persistent organic pollutant; SRS-2, Social Responsiveness Scale-2; β-HCH, β-Hexachlorocyclohexane.

Table S4. Adjusted[a] associations between chemical mixtures and SRS-2 T-scores using quantile g-computation and **assessing modification by gestational folic acid supplementation**, including quantile g-computation weights, the MIREC study, Canada, 2008-2011 (n = 601).

Mixture name	Metals			OC Pesticides			PFAS			PCBs			All POPs		
FA supplementation[b] (µg/day)	<400	400-1000	>1000	<400	400-1000	>1000	<400	400-1000	>1000	<400	400-1000	>1000	<400	400-1000	>1000
Ψ (95% CI)[a]	0.6 (-1.6, 2.8)	-0.2 (-1.1, 0.7)	2.4 (0.8, 3.9)	1.3 (-0.8, 3.3)	-0.3 (-1.1, 0.5)	0.6 (-0.7, 1.8)	1.4 (-0.8, 3.6)	-0.8 (-1.4, -0.1)	-0.3 (-1.3, 0.8)	1.9 (0.0, 3.8)	-0.1 (-0.8, 0.6)	1.0 (0.0, 2.0)	2.9 (-0.8, 6.6)	-0.6 (-1.8, 0.5)	0.7 (-1.4, 2.7)
Biomarker names	Weights[c]														
Arsenic	0.4	-0.35	0.17												
Cadmium	-0.55	0.47	0.08												
Lead	0.6	0.53	0.42												
Mercury	-0.45	-0.65	0.33												
β-HCH				-0.19	-0.39	0.28							0.01	-0.03	-0.09
DDE				0.82	-0.05	0.69							-0.12	-0.08	-0.04
Oxychlordane				0.18	-0.56	0.03							-0.12	-0.06	0.01
trans-Nonachlor				-0.81	1	-1							-0.22	0.03	-0.22
PFHxS							-0.27	1	0.47				-0.11	0.28	0.1
PFOS							1	-0.77	-1				0.17	-0.37	-0.19
PFOA							-0.73	-0.23	0.53				0	-0.11	-0.01
PCB118										0.12	-0.62	0.19	0.1	-0.17	0.16
PCB138										-0.44	1	-0.55	-0.4	0.55	-0.21
PCB153										0.88	-0.36	0.81	0.55	-0.13	0.62
PCB180										-0.56	-0.02	-0.45	-0.03	-0.05	-0.25
BDE47													0.17	0.14	0.1

a: Controls for the following variables: child sex, child age at SRS-2 assessment, HOME score, household income, relationship status, maternal education, maternal race, maternal age, parity, smoking status, city of residence, and year of enrollment. Effect estimates are pooled across 10 multiply imputed datasets. Stabilized inverse probability weights are applied.

b: Folic acid supplementation was primarily measured via a survey conducted at 16 weeks gestation, which queried intake in the past 30 days. We also used data from the 24-hour recall version of this survey and a questionnaire completed at study enrollment (6-13 weeks gestation).

c: Only the weights from the first MICE imputation, not the pooled weights, are displayed.

Abbreviations: BDE, brominated diphenyl ether; CI; Confidence interval; DDE, Dichlorodiphenyldichloroethylene; HOME, Home Observation for Measurement of the Environment; MICE, Multiple imputation by chained equations; MIREC, Maternal-Infant Research on Environmental Chemicals Study; OC, Organochlorine; PCB, Polychlorinated biphenyl; PFAS, Per- and polyfluoroalkyl substances; PFHxS, Perfluorohexanesulfonic acid; PFOA, Perfluorooctanoic acid; PFOS, Perfluorooctanesulfonic acid; POP, Persistent organic pollutant; SRS-2, Social Responsiveness Scale-2; β-HCH, β-Hexachlorocyclohexane.

Table S5. Adjusted[a] associations between chemical mixtures and SRS-2 T-scores using quantile g-computation and **assessing modification by plasma total folate concentrations**, including quantile g-computation weights, the MIREC study, Canada, 2008-2011 (n = 601).

Mixture name	Metals			OC Pesticides			PFAS			PCBs			All POPs		
Plasma total folate concentration[b]	<10th %ile	10th-80th %ile	>80th %ile	<10th %ile	10th-80th %ile	>80th %ile	<10th %ile	10th-80th %ile	>80th %ile	<10th %ile	10th-80th %ile	>80th %ile	<10th %ile	10th-80th %ile	>80th %ile
Ψ (95% CI)[a]	-0.1 (-3.1, 2.8)	-0.1 (-1.0, 0.8)	1.9 (0.4, 3.3)	0.2 (-1.7, 2.1)	0.0 (-0.8, 0.7)	0.4 (-0.9, 1.7)	-0.5 (-2.4, 1.5)	-0.3 (-0.9, 0.4)	-1.5 (-2.6, -0.3)	0.4 (-1.4, 2.2)	0.4 (-0.2, 1.0)	0.3 (-0.9, 1.5)	1.1 (-1.7, 4.0)	0.2 (-0.9, 1.4)	-1.9 (-4.3, 0.6)
Biomarker names	Weights[c]														
Arsenic	0.02	-0.15	-1												
Cadmium	-0.4	-0.06	0.5												
Lead	0.98	1	0.42												
Mercury	-0.6	-0.79	0.07												
β-HCH				-0.18	-0.29	0.29							-0.08	-0.12	0.04
DDE				0.25	1	-1							-0.05	-0.01	-0.21
Oxychlordane				-0.82	-0.14	0.71							-0.51	-0.11	0.31
trans-Nonachlor				0.75	-0.57	0							0.4	-0.28	-0.04
PFHxS							-1	1	1				-0.19	0.33	0.19
PFOS							0.97	-0.74	-0.87				0.04	-0.32	-0.36
PFOA							0.03	-0.26	-0.13				0.04	-0.11	-0.08
PCB118										-0.35	-0.53	0.24	-0.16	-0.05	0.26
PCB138										-0.42	0.87	0.29	0.08	0.43	0.13
PCB153										1	0.13	0.47	0.2	0.07	0.07
PCB180										-0.23	-0.47	-1	0	0	-0.19
BDE47													0.24	0.18	-0.12

a: Controls for the following variables: child sex, child age at SRS-2 assessment, HOME score, household income, relationship status, maternal education, maternal race, maternal age, parity, smoking status, city of residence, and year of enrollment. Effect estimates are pooled across 10 multiply imputed datasets. Stabilized inverse probability weights are applied.
b: The sum of 5-formyl-THF, 5-10-methylene-THF, THF, UMFA, and 5-methyl-THF.
c: Only the weights from the first MICE imputation, not the pooled weights, are displayed.
Abbreviations: BDE, brominated diphenyl ether; CI; Confidence interval; DDE, Dichlorodiphenyldichloroethylene; HOME, Home Observation for Measurement of the Environment; MICE, Multiple imputation by chained equations; MIREC, Maternal-Infant Research on Environmental Chemicals Study; OC, Organochlorine; PCB, Polychlorinated biphenyl; PFAS, Per- and polyfluoroalkyl substances; PFHxS, Perfluorohexanesulfonic acid; PFOA, Perfluorooctanoic acid; PFOS, Perfluorooctanesulfonic acid; POP, Persistent organic pollutant; THF, Tetrahydrofolate; UMFA, Unmetabolized folic acid; SRS-2, Social Responsiveness Scale-2; β-HCH, β-Hexachlorocyclohexane; %ile, Percentile.

Table S6. Adjusted[a] associations between chemical mixtures and SRS-2 T-scores **using weighted quantile sum regression**, including weighted quantile sum weights, the MIREC study, Canada, 2008-2011 (n = 601).

Mixture name	Metals		OC Pesticides		PFAS		PCBs		All POPs	
Partial effect	(+)	(-)	(+)	(-)	(+)	(-)	(+)	(-)	(+)	(-)
β (95% CI)[a]	0.5 (-0.2, 1.2)	0.6 (-0.2, 1.4)	0.3 (-0.5, 1.1)	0.4 (-0.4, 1.2)	-0.5 (-1.1, 0.0)	-1.0 (-1.7, -0.3)	0.8 (0.1, 1.4)	0.8 (0.2, 1.5)	-0.1 (-1.0, 0.9)	0.3 (-0.7, 1.3)
Biomarker names	Weights[b]									
Arsenic	0.02	0.36								
Cadmium	0.72	0.05								
Lead	0.25	0.11								
Mercury	0.01	0.47								
β-HCH			0.14	0.42					0.01	0.25
DDE			0.25	0.16					0.01	0.07
Oxychlordane			0.06	0.38					0	0.16
trans-Nonachlor			0.55	0.04					0.03	0.01
PFHxS					0.96	0			0.44	0
PFOS					0.01	0.6			0	0.13
PFOA					0.04	0.4			0.01	0.11
PCB118							0.24	0.41	0.01	0.1
PCB138							0.03	0.34	0	0.06
PCB153							0.48	0.02	0.04	0
PCB180							0.25	0.23	0.05	0.1
BDE47									0.39	0

a: Controls for the following variables: child sex, gestational folic acid supplementation, child age at SRS-2 assessment, HOME score, household income, relationship status, maternal education, maternal race, maternal age, parity, smoking status, city of residence, and year of enrollment. Effect estimates are pooled across 10 multiply imputed datasets. Stabilized inverse probability weights are applied.

b: Weights are averaged across 10 multiply imputed datasets.

Abbreviations: BDE, brominated diphenyl ether; CI; Confidence interval; DDE, Dichlorodiphenyldichloroethylene; HOME, Home Observation for Measurement of the Environment; MICE, Multiple imputation by chained equations; MIREC, Maternal-Infant Research on Environmental Chemicals Study; OC, Organochlorine; PCB, Polychlorinated biphenyl; PFAS, Per- and polyfluoroalkyl substances; PFHxS, Perfluorohexanesulfonic acid; PFOA, Perfluorooctanoic acid; PFOS, Perfluorooctanesulfonic acid; POP, Persistent organic pollutant; SRS-2, Social Responsiveness Scale-2; β-HCH, β-Hexachlorocyclohexane.

Table S7. Adjusted[a] associations between chemical mixtures and SRS-2 T-scores using quantile g-computation and **assessing modification by plasma unmetabolized folic acid concentrations**, including quantile g-computation weights, the MIREC study, Canada, 2008-2011 (n = 601).

Mixture name	Metals		OC Pesticides		PFAS		PCBs		All POPs	
Plasma unmetabolized folic acid concentrations	≤80th %ile	>80th %ile	≤80th %ile	>80th %ile	≤80th %ile	>80th %ile	≤80th %ile	>80th %ile	≤80th %ile	>80th %ile
Ψ (95% CI)[a]	0.2 (-0.7, 1.1)	1.0 (-0.6, 2.6)	0.0 (-0.7, 0.7)	0.4 (-0.9, 1.7)	-0.3 (-0.9, 0.3)	-1.2 (-2.3, 0.0)	0.4 (-0.2, 1.0)	0.5 (-0.8, 1.7)	0.3 (-0.8, 1.3)	-0.6 (-2.9, 1.7)
Biomarker names	Weights[b]									
Arsenic	-0.33	0.25								
Cadmium	0.16	0.21								
Lead	0.84	0.27								
Mercury	-0.67	0.27								
β-HCH			-0.45	0.23					-0.14	0.12
DDE			0.86	0.36					-0.09	0.08
Oxychlordane			0.14	-1					-0.01	-0.25
trans-Nonachlor			-0.55	0.41					-0.28	0.11
PFHxS					1	1			0.25	0.16
PFOS					-0.85	-0.26			-0.38	-0.08
PFOA					-0.15	-0.74			-0.05	-0.32
PCB118							-0.54	-0.47	-0.04	-0.03
PCB138							0.25	0.73	0.14	0.31
PCB153							0.75	0.27	0.32	0.22
PCB180							-0.46	-0.53	0.01	-0.27
BDE47									0.28	-0.05

a: Controls for the following variables: child sex, child age at SRS-2 assessment, HOME score, household income, relationship status, maternal education, maternal race, maternal age, parity, smoking status, city of residence, and year of enrollment. Effect estimates are pooled across 10 multiply imputed datasets. Stabilized inverse probability weights are applied.
b: Only the weights from the first MICE imputation, not the pooled weights, are displayed.
Abbreviations: BDE, brominated diphenyl ether; CI; Confidence interval; DDE, Dichlorodiphenyldichloroethylene; HOME, Home Observation for Measurement of the Environment; MICE, Multiple imputation by chained equations; MIREC, Maternal-Infant Research on Environmental Chemicals Study; OC, Organochlorine; PCB, Polychlorinated biphenyl; PFAS, Per- and polyfluoroalkyl substances; PFHxS, Perfluorohexanesulfonic acid; PFOA, Perfluorooctanoic acid; PFOS, Perfluorooctanesulfonic acid; POP, Persistent organic pollutant; SRS-2, Social Responsiveness Scale-2; β-HCH, β-Hexachlorocyclohexane; %ile, Percentile.

Table S8. Adjusted[a] associations (**additionally controlling for gestational fish consumption**) between chemical mixtures and SRS-2 T-scores using quantile g-computation, including quantile g-computation weights, the MIREC study, Canada, 2008-2011 (n = 601).

Mixture name	Metals	OC Pesticides	PFAS	PCBs	All POPs
Ψ (95% CI)[a]	0.3 (-0.5, 1.1)	0.0 (-0.6, 0.7)	-0.5 (-1.1, 0.1)	0.4 (-0.2, 1.0)	0.1 (-0.9, 1.1)
Biomarker names	Weights[b]				
Arsenic	-0.23				
Cadmium	0.25				
Lead	0.75				
Mercury	-0.77				
β-HCH		-0.32			-0.07
DDE		1			-0.05
Oxychlordane		-0.4			-0.07
trans-Nonachlor		-0.28			-0.17
PFHxS			1		0.22
PFOS			-0.71		-0.34
PFOA			-0.29		-0.15
PCB118				-0.47	-0.03
PCB138				0.46	0.27
PCB153				0.54	0.3
PCB180				-0.53	-0.12
BDE47					0.21

a: Controls for the following variables: gestational fish consumption, child sex, gestational folic acid supplementation, child age at SRS-2 assessment, HOME score, household income, relationship status, maternal education, maternal race, maternal age, parity, smoking status, city of residence, and year of enrollment. Effect estimates are pooled across 10 multiply imputed datasets. Stabilized inverse probability weights are applied.
b: Only the weights from the first MICE imputation, not the pooled weights, are displayed.
Abbreviations: BDE, brominated diphenyl ether; CI; Confidence interval; DDE, Dichlorodiphenyldichloroethylene; HOME, Home Observation for Measurement of the Environment; MICE, Multiple imputation by chained equations; MIREC, Maternal-Infant Research on Environmental Chemicals Study; OC, Organochlorine; PCB, Polychlorinated biphenyl; PFAS, Per- and polyfluoroalkyl substances; PFHxS, Perfluorohexanesulfonic acid; PFOA, Perfluorooctanoic acid; PFOS, Perfluorooctanesulfonic acid; POP, Persistent organic pollutant; SRS-2, Social Responsiveness Scale-2; β-HCH, β-Hexachlorocyclohexane.

Table S9. Adjusted[a] associations between chemical mixtures and SRS-2 T-scores using quantile g-computation **without inverse probability weighting**, the MIREC study, Canada, 2008-2011 (n = 601).

Mixture name	Metals	OC Pesticides	PFAS	PCBs	All POPs
Ψ (95% CI)[a]	-0.1<br>(-0.8, 0.7)	-0.2<br>(-0.9, 0.4)	-0.6<br>(-1.2, 0.0)	0.0<br>(-0.6, 0.6)	-0.5<br>(-1.5, 0.4)
Biomarker names	Weights[b]				
Arsenic	-0.19				
Cadmium	0.37				
Lead	0.63				
Mercury	-0.81				
β-HCH		-0.5			-0.12
DDE		0.7			-0.07
Oxychlordane		-0.5			-0.08
trans-Nonachlor		0.3			-0.07
PFHxS			1		0.29
PFOS			-0.48		-0.22
PFOA			-0.52		-0.21
PCB118				-0.57	-0.08
PCB138				0.66	0.38
PCB153				0.34	0.19
PCB180				-0.43	-0.15
BDE47					0.15

a: Controls for the following variables: child sex, gestational folic acid supplementation, child age at SRS-2 assessment, HOME score, household income, relationship status, maternal education, maternal race, maternal age, parity, smoking status, city of residence, and year of enrollment. Effect estimates are pooled across 10 multiply imputed datasets. Stabilized inverse probability weights are not applied.

b: Only the weights from the first MICE imputation, not the pooled weights, are displayed.

Abbreviations: BDE, brominated diphenyl ether; CI; Confidence interval; DDE, Dichlorodiphenyldichloroethylene; HOME, Home Observation for Measurement of the Environment; MICE, Multiple imputation by chained equations; MIREC, Maternal-Infant Research on Environmental Chemicals Study; OC, Organochlorine; PCB, Polychlorinated biphenyl; PFAS, Per- and polyfluoroalkyl substances; PFHxS, Perfluorohexanesulfonic acid; PFOA, Perfluorooctanoic acid; PFOS, Perfluorooctanesulfonic acid; POP, Persistent organic pollutant; SRS-2, Social Responsiveness Scale-2; β-HCH, β-Hexachlorocyclohexane.

Table S10. Adjusted[a] associations between chemical mixtures and SRS-2 T-scores using quantile g-computation and assessing modification by gestational folic acid supplementation **without inverse probability weighting**, the MIREC study, Canada, 2008-2011 (n = 601).

Mixture name	Metals			OC Pesticides			PFAS			PCBs			All POPs		
FA supplementation[b] (µg/day)	<400	400-1000	>1000	<400	400-1000	>1000	<400	400-1000	>1000	<400	400-1000	>1000	<400	400-1000	>1000
Ψ (95% CI)[a]	-0.3 (-2.7, 2.0)	-0.6 (-1.4, 0.3)	1.7 (0.1, 3.3)	0.4 (-1.8, 2.7)	-0.5 (-1.3, 0.2)	0.3 (-0.9, 1.6)	0.4 (-1.9, 2.6)	-0.9 (-1.6, -0.2)	-0.2 (-1.3, 0.9)	1.1 (-0.7, 3.0)	-0.2 (-0.9, 0.4)	0.3 (-0.7, 1.4)	2.1 (-2.2, 6.3)	-1.0 (-2.0, 0.1)	0.0 (-2.1, 2.0)
Biomarker names	Weights[c]														
Arsenic	0.45	-0.31	0.11												
Cadmium	-0.55	0.19	0.31												
Lead	0.55	0.81	0.2												
Mercury	-0.45	-0.69	0.38												
β-HCH				-0.09	-0.54	0.01							0.06	-0.1	-0.02
DDE				0.78	1	0.12							-0.17	-0.04	-0.02
Oxychlordane				0.22	-0.24	-1							-0.1	-0.02	-0.21
trans-Nonachlor				-0.91	-0.22	0.87							-0.17	-0.11	0.24
PFHxS							-0.59	1	1				-0.17	0.35	0.11
PFOS							1	-0.62	-0.46				0.22	-0.31	-0.13
PFOA							-0.41	-0.38	-0.54				0.01	-0.16	-0.12
PCB118										0	-0.67	0.08	-0.03	-0.12	-0.01
PCB138										0.52	1	-0.58	-0.04	0.53	-0.2
PCB153										0.48	-0.12	0.92	0.55	-0.04	0.6
PCB180										-1	-0.21	-0.42	-0.31	-0.09	-0.29
BDE47													0.16	0.12	0.05

a: Controls for the following variables: child sex, child age at SRS-2 assessment, HOME score, household income, relationship status, maternal education, maternal race, maternal age, parity, smoking status, city of residence, and year of enrollment. Effect estimates are pooled across 10 multiply imputed datasets. Stabilized inverse probability weights are not applied.
b: Folic acid supplementation was primarily measured via a survey conducted at 16 weeks gestation, which queried intake in the past 30 days. We also used data from the 24-hour recall version of this survey and a questionnaire completed at study enrollment (6-13 weeks gestation).
c: Only the weights from the first MICE imputation, not the pooled weights, are displayed.
Abbreviations: BDE, brominated diphenyl ether; CI; Confidence interval; DDE, Dichlorodiphenyldichloroethylene; HOME, Home Observation for Measurement of the Environment; MICE, Multiple imputation by chained equations; MIREC, Maternal-Infant Research on Environmental Chemicals Study; OC, Organochlorine; PCB, Polychlorinated biphenyl; PFAS, Per- and polyfluoroalkyl substances; PFHxS, Perfluorohexanesulfonic acid; PFOA, Perfluorooctanoic acid; PFOS, Perfluorooctanesulfonic acid; POP, Persistent organic pollutant; SRS-2, Social Responsiveness Scale-2; β-HCH, β-Hexachlorocyclohexane.

Table S11. Adjusted[a] associations between chemical mixtures and SRS-2 T-scores using quantile g-computation and assessing modification by plasma total folate concentrations **without inverse probability weighting**, the MIREC study, Canada, 2008-2011 (n = 601).

Mixture name	Metals			OC Pesticides			PFAS			PCBs			All POPs		
Plasma total folate concentration[b]	<10th %ile	10th-80th %ile	>80th %ile	<10th %ile	10th-80th %ile	>80th %ile	<10th %ile	10th-80th %ile	>80th %ile	<10th %ile	10th-80th %ile	>80th %ile	<10th %ile	10th-80th %ile	>80th %ile
Ψ (95% CI)[a]	-0.7 (-3.2, 1.8)	-0.3 (-1.2, 0.6)	1.1 (-0.3, 2.6)	0.0 (-1.9, 1.9)	-0.3 (-1.0, 0.4)	0.2 (-1.1, 1.5)	-1.0 (-2.8, 0.8)	-0.5 (-1.2, 0.1)	-1.3 (-2.6, 0.0)	0.4 (-1.4, 2.1)	0.0 (-0.7, 0.6)	0.1 (-1.0, 1.2)	0.2 (-2.4, 2.9)	-0.5 (-1.6, 0.7)	-1.6 (-4.0, 0.9)
Biomarker names	Weights[c]														
Arsenic	0.44	-0.2	-0.59												
Cadmium	-0.33	1	0.49												
Lead	0.56	-0.46	0.51												
Mercury	-0.67	-0.34	-0.41												
β-HCH				-0.24	-0.6	0.75							-0.07	-0.15	0.28
DDE				0.22	1	-0.73							-0.12	-0.02	-0.18
Oxychlordane				-0.76	-0.12	0.25							-0.36	-0.03	0.02
trans-Nonachlor				0.78	-0.28	-0.27							0.3	-0.16	-0.02
PFHxS							-0.47	1	1				-0.18	0.32	0.16
PFOS							1	-0.54	-0.75				0.04	-0.22	-0.39
PFOA							-0.53	-0.46	-0.25				0	-0.16	-0.15
PCB118										-0.5	-0.57	0.67	-0.21	-0.11	0.19
PCB138										-0.25	0.8	0.33	0.1	0.49	0.31
PCB153										1	0.2	-0.74	0.34	0.12	-0.06
PCB180										-0.25	-0.43	-0.26	-0.07	-0.16	-0.2
BDE47													0.22	0.07	0.05

a: Controls for the following variables: child sex, child age at SRS-2 assessment, HOME score, household income, relationship status, maternal education, maternal race, maternal age, parity, smoking status, city of residence, and year of enrollment. Effect estimates are pooled across 10 multiply imputed datasets. Stabilized inverse probability weights are not applied.
b: The sum of 5-formyl-THF, 5-10-methylene-THF, THF, UMFA, and 5-methyl-THF.
c: Only the weights from the first MICE imputation, not the pooled weights, are displayed.
Abbreviations: BDE, brominated diphenyl ether; CI; Confidence interval; DDE, Dichlorodiphenyldichloroethylene; HOME, Home Observation for Measurement of the Environment; MICE, Multiple imputation by chained equations; MIREC, Maternal-Infant Research on Environmental Chemicals Study; OC, Organochlorine; PCB, Polychlorinated biphenyl; PFAS, Per- and polyfluoroalkyl substances; PFHxS, Perfluorohexanesulfonic acid; PFOA, Perfluorooctanoic acid; PFOS, Perfluorooctanesulfonic acid; POP, Persistent organic pollutant; THF, Tetrahydrofolate; UMFA, Unmetabolized folic acid; SRS-2, Social Responsiveness Scale-2; β-HCH, β-Hexachlorocyclohexane; %ile, Percentile.

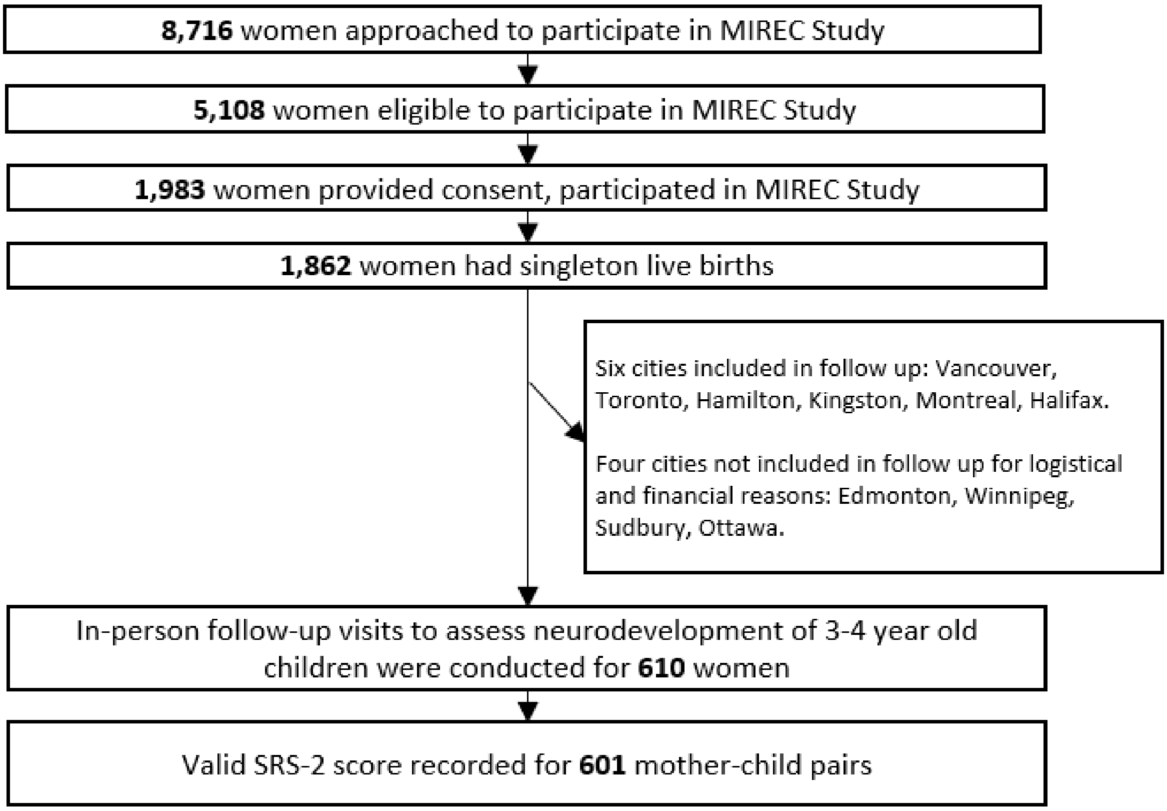

Figure S1. Flow chart of study participants in sample, the MIREC Study, Canada, 2008-11.
Abbreviations: MIREC, Maternal-Infant Research on Environmental Chemicals Study; SRS-2, Social Responsiveness Scale-2.

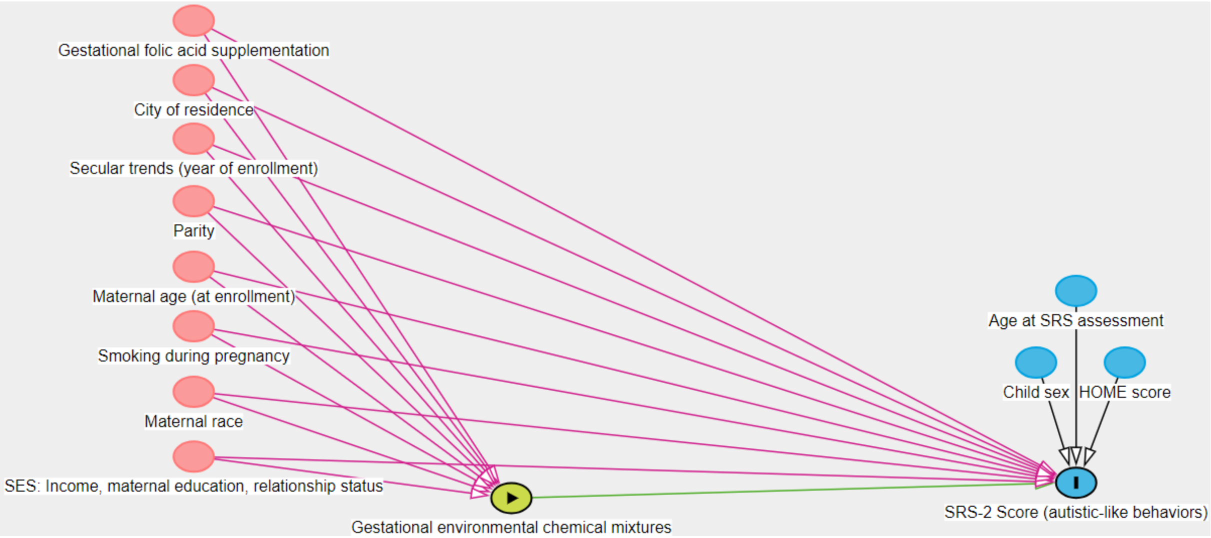

Figure S2. Directed acyclic graph depicting our assumptions about the relationship between gestational environmental chemical mixtures and autistic-like behaviors.

Abbreviations: HOME, Home Observation for Measurement of the Environment; SES, socioeconomic status; SRS-2, Social Responsiveness Scale-2.

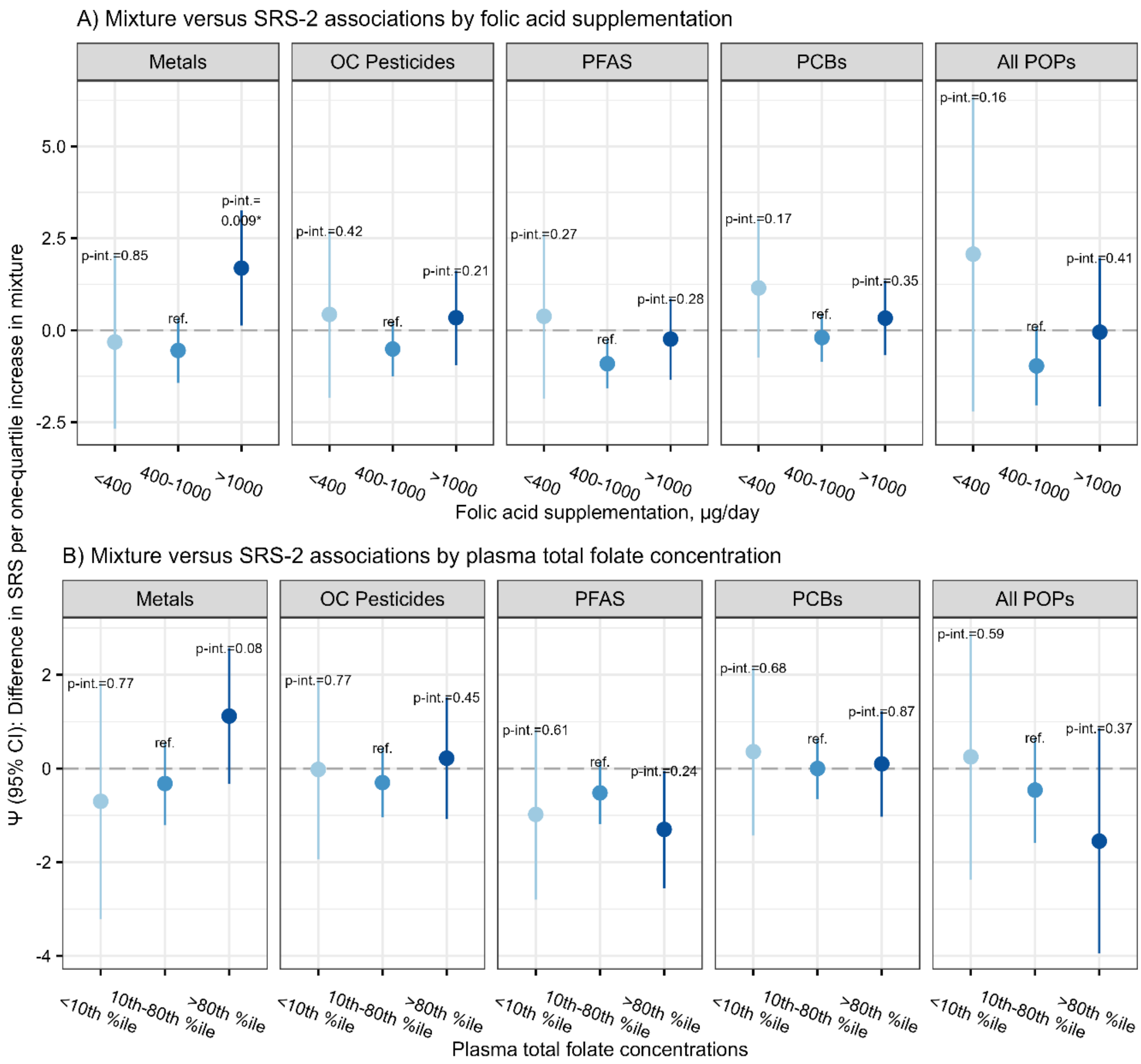

Figure S3. Adjusted associations between chemical mixtures and child SRS-2 T-scores using quantile g-computation and assessing interaction by gestational folic acid supplementation and plasma total folate concentrations, without inverse probability weighting, the MIREC study, Canada, 2008-2011 (n = 601). Controls for the following variables: child sex, child age at SRS-2 assessment, HOME score, household income, relationship status, maternal education, maternal race, maternal age, parity, smoking status, city of residence, and year of enrollment. Plot A additionally controls for folic acid supplementation. Effect estimates are pooled across 10 multiply imputed datasets. The metal mixture includes arsenic, cadmium, lead, and mercury. The OC pesticides mixtures includes β-HCH, DDE, oxychlordane, and trans-nonachlor. The PFAS mixture includes PFHxS, PFOS, and PFOA. The PCB mixture includes PCB118, PCB138, PCB153, and PCB180. The POP mixture includes β-HCH, DDE, oxychlordane, trans-nonachlor, PFHxS, PFOS, PFOA, PCB118, PCB138, PCB153, PCB180, and BDE47.

*: Denotes an interaction P-value (i.e., the P-value of the interaction term) less than 0.05. All P-values are two-sided.

Abbreviations: BDE, brominated diphenyl ether; DDE, Dichlorodiphenyldichloroethylene; HOME, Home Observation for Measurement of the Environment; MIREC, Maternal-Infant Research on Environmental

Chemicals Study; PCB, Polychlorinated biphenyl; PFHxS, Perfluorohexanesulfonic acid; PFOA, Perfluorooctanoic acid; PFOS, Perfluorooctanesulfonic acid; p-int, P-interaction; SRS-2, Social Responsiveness Scale-2; UMFA, Unmetabolized folic acid; β-HCH, β-Hexachlorocyclohexane; %ile, Percentile.

References

1. White IR, Royston P, Wood AM. Multiple imputation using chained equations: Issues and guidance for practice. *Stat Med*. 2011;30(4):377-399.

2. van Buuren S, Groothuis-Oudshoorn K. mice: Multivariate Imputation by Chained Equations in R. *J Stat Softw*. 2011;45:1-67.

3. Keil AP, Buckley JP, O'Brien KM, Ferguson KK, Zhao S, White AJ. A quantile-based g-computation approach to addressing the effects of exposure mixtures. *Environ Health Perspect*. 2020;128(4):47004.

4. Carrico C, Gennings C, Wheeler DC, Factor-Litvak P. Characterization of Weighted Quantile Sum Regression for Highly Correlated Data in a Risk Analysis Setting. *J Agric Biol Environ Stat*. 2015;20(1):100-120.

5. Hernán MA, Hernández-Díaz S, Robins JM. A structural approach to selection bias. *Epidemiology*. 2004;15(5):615-625.
